# Multi‐omics data mining combined with experimental validation reveals ferroptosis‐ and autophagy‐associated hub genes as diagnostic candidates and immune modulators in atherosclerosis

**DOI:** 10.1002/ame2.70208

**Published:** 2026-05-06

**Authors:** Xinou Zheng, Jinling Zheng, Xuezhuang Li, Hua Chen, Li Zhang, Yuqiong Zhao, Yahao Ling

**Affiliations:** ^1^ Medical Innovation Research Department Chinese PLA General Hospital Beijing China; ^2^ College of Medical Technology Beihua University Jilin City Jilin Province China; ^3^ Beijing Engineering Research Center for Experimental Animal Models of Human Diseases, Institute of Laboratory Animal Science Peking Union Medicine College, Chinese Academy of Medical Sciences Beijing China; ^4^ Department of Pharmacy People's Hospital of Longhua Shenzhen Guangdong China

**Keywords:** atherosclerosis, autophagy, bioinformatics analysis, biomarkers, ferroptosis, machine learning

## Abstract

**Background:**

Atherosclerosis (AS) is a chronic inflammatory vascular disease that can lead to severe cardiovascular events. Ferroptosis and autophagy have been increasingly recognized for their significant roles in AS; however, few clinically translatable hub genes that connect these processes in atherosclerotic lesions have been identified. There is an urgent need for novel diagnostic and therapeutic targets to improve the early detection and intervention of AS.

**Methods:**

We downloaded AS‐related datasets from the Gene Expression Omnibus (GEO) database. Common genes were identified using Limma and Weighted Gene Coexpression Network Analysis (WGCNA). We assessed ferroptosis and autophagy marker expression in AS mouse coronary tissues using immunohistochemistry (IHC) and immunofluorescence (IF). Hub genes were identified by intersecting common genes with known ferroptosis‐ and autophagy‐related gene sets. Gene Set Enrichment Analysis (GSEA), receiver operating characteristic (ROC) curves, and 10‐fold cross‐validation repeated five times were performed to explore the potential roles and diagnostic capabilities of these hub genes. Immune cell infiltration analysis was performed using CIBERSORT, and Spearman's correlation analysis was subsequently performed to evaluate the associations between the identified hub genes and the relative abundance of immune cells. Finally, hub gene expression was validated in AS mouse coronary tissues using IHC and IF.

**Results:**

Limma and WGCNA identified 104 common genes. IHC and IF analyses in AS mice confirmed the activation of ferroptosis and inhibition of autophagy in the coronary tissue. Intersecting common genes with pathway‐specific genes identified three pivotal hub genes: CALCOCO2, TXNRD1, and SELENBP1. ROC analysis and 10‐fold cross‐validation repeated five times indicated excellent diagnostic efficacy for these genes. Immune infiltration analysis revealed significant alterations in immune cell populations in AS patients, and correlation analysis further demonstrated that CALCOCO2, TXNRD1, and SELENBP1 were significantly associated with multiple immune cell types, directly linking these hub genes to the atherosclerotic immune microenvironment. Consistent with patient data, IHC/IF validation showed significantly lower expression of the hub genes in AS mouse coronary tissues compared to controls.

**Conclusions:**

CALCOCO2, TXNRD1, and SELENBP1 represent a novel panel of diagnostic biomarkers uniquely positioned at the intersection of ferroptosis and autophagy in AS. The robust diagnostic performance of these hub genes, as demonstrated by ROC analysis and cross‑validation, was further consolidated by IHC/IF validation showing their consistently downregulated expression in AS mouse models. Furthermore, these hub genes are significantly correlated with multiple immune cell populations, directly linking them to the atherosclerotic immune microenvironment and reinforcing their role as immune modulators in AS pathogenesis. Beyond diagnosis, these hub genes illuminate clinically actionable therapeutic targets: restoring their expression or function may simultaneously correct autophagy dysfunction and suppress ferroptosis, offering a dual‐mechanism intervention strategy for atherosclerotic cardiovascular disease. Our findings provide a molecular framework for precision medicine approaches targeting ferroptosis–autophagy crosstalk and immune dysregulation in AS.

## Graphical abstract

Using the GSE225650 dataset, we performed differential expression gene (DEG) screening, Weighted Gene Coexpression Network Analysis (WGCNA), and immune infiltration profiling on atherosclerosis (AS) samples, yielding 104 shared candidate genes. Subsequently, these 104 candidate genes were intersected with ferroptosis‑ and autophagy‑related gene sets using a Venn diagram, yielding three hub genes: CALCOCO2, TXNRD1, and SELENBP1. Subsequent validation using Gene Set Enrichment Analysis (GSEA), receiver operating characteristic (ROC) curve analysis, patient expression validation, and in vivo mouse tissue assays confirmed these genes as potential biomarkers bridging ferroptosis and autophagy in AS pathogenesis.

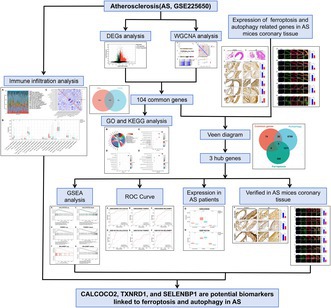

## 1 INTRODUCTION

Atherosclerosis (AS) is a multifocal, chronic immunoinflammatory disease of medium‐sized and large arteries, primarily driven by lipid accumulation.^1^ It is characterized by intra‐arterial plaque formation rich in cells and lipids.^2^ Plaque progression leads to arterial wall stiffening, luminal narrowing, and potential plaque destabilization or rupture. These events can precipitate severe clinical outcomes, including myocardial infarction, ischemic stroke, and peripheral artery disease, which are leading causes of global mortality.^3^ Indeed, cardiovascular diseases were responsible for 20.5 million deaths worldwide in 2021, representing one‐third of all fatalities, according to the 2023 World Heart Report released by the World Heart Federation.^4^ Current AS diagnosis predominantly relies on imaging techniques like ultrasound, magnetic resonance, and computed tomography. However, these modalities are limited to identifying advanced lesions and lack the sensitivity to accurately assess plaque vulnerability.^5^ Biomarkers are routinely used in clinical medicine for disease diagnosis, prognostication, guiding ongoing clinical decisions, and monitoring therapeutic efficacy.^6^ Therefore, identifying reliable biomarkers for AS is crucial for facilitating early diagnosis and intervention.

Ferroptosis, a distinct form of programmed cell death first described by Dixon et al.^7^ in 2012, is characterized by iron‐dependent accumulation of reactive oxygen species (ROS) and lethal lipid peroxides (LPO). The core mechanism involves an imbalance in cellular redox homeostasis. It was shown that glutathione peroxidase 4 (GPX4) overexpression in apolipoprotein e‐deficient (*Apoe* ^−/−^) mice inhibits AS progression by reducing lipid peroxidation and the susceptibility of vascular cells to oxidized lipids.^8^ More recently, Luo et al.^9^ demonstrated that Micheliolide suppresses AS by inhibiting macrophage ferroptosis via NRF2 pathway activation, competitively binding to the Arg483 site of KEAP1. Collectively, these findings underscore the critical role of ferroptosis in the development and progression of AS, suggesting that ferroptosis‐associated biomarkers could significantly aid in early diagnosis of AS.

Autophagy, a highly conserved cellular recycling process, enables eukaryotes to maintain cellular and organismal homeostasis by degrading intracellular components or selectively removing damaged organelles.^10^ Its involvement in AS is also well documented. Yu et al.^11^ revealed that Leonurine exerts anti‐atherosclerotic effects by activating autophagy through enhanced METTL3‐mediated AKT1S1 stability. However, the role of autophagy in atherosclerosis is complex and context dependent. Although basal autophagy is generally protective in vascular cells—promoting endothelial survival,^12^ maintaining vascular smooth muscle cell (VSMC) homeostasis,^13^ and facilitating cholesterol efflux from macrophage foam cells^14^—dysregulated or excessive autophagy can be detrimental. For instance, hyperactivation of autophagy has been linked to VSMC death and plaque destabilization,^15^ whereas impaired autophagic flux in macrophages may exacerbate inflammation and lipid accumulation.^16^, ^17^ Thus, the net effect of autophagy on plaque progression depends critically on the cellular context and the stage of the disease.

It is reported that ferroptosis and autophagy are not isolated cellular events; instead, they interact bidirectionally to regulate cell survival and death in various diseases, including AS. Accumulating evidence has shown that autophagy activation is crucial for inhibiting macrophage ferroptosis and alleviating atherosclerosis.^18^, ^19^ Specifically, autophagy plays a pivotal role in regulating ferroptosis by modulating intracellular iron homeostasis and ROS generation,^20^ and it also facilitates lipid hydrolysis and cholesterol efflux in macrophage‐derived foam cells to delay lipid accumulation.^21^ However, the specific role and molecular mechanism of this ferroptosis‐autophagy crosstalk in AS remain incompletely elucidated, and the potential link mediated by their core markers (GPX4 for ferroptosis and LC3 for autophagy) has not been fully explored. Against this background, the colocalization of GPX4 and LC3 observed in our preliminary experiments, combined with their potential abnormal expression in AS, provides a unique opportunity to explore the functional crosstalk between ferroptosis and autophagy in atherosclerotic lesions—laying the foundation for our subsequent identification of hub genes associated with both processes.

The rapid advancement of transcriptomics has facilitated the use of bioinformatics analyses of gene expression profile data to identify signature biomarkers.^22^, ^23^ In this study, we leveraged public datasets from the Gene Expression Omnibus (GEO) to identify differentially expressed genes (DEGs) between AS and normal samples. We then employed Weighted Gene Coexpression Network Analysis (WGCNA) to identify key gene modules associated with AS. By intersecting DEGs with genes from these key modules, we identified a set of common genes whose biological functions were explored using Gene Ontology (GO) and Kyoto Encyclopedia of Genes and Genomes (KEGG) pathway analyses. To further investigate the roles of ferroptosis and autophagy in AS, we examined the protein expression of respective markers in coronary artery tissues from an AS mouse model. Immunofluorescence (IF) colocalization suggested a close relationship between autophagy and ferroptosis in AS. Consequently, we identified hub genes by intersecting the common genes with established ferroptosis‐ and autophagy‐related gene sets. Gene Set Enrichment Analysis (GSEA) was then performed to elucidate the potential roles of these hub genes, and their diagnostic efficacy was evaluated using the receiver operating characteristic (ROC) curve. We also analyzed immune cell infiltration patterns using the CIBERSORT algorithm, and subsequently performed Spearman's correlation analysis to evaluate the associations between the identified hub genes and the relative abundance of immune cells. Finally, the expression of identified hub genes was validated in the coronary artery tissues from AS mouse models. This study aimed to identify and validate biomarkers linked to ferroptosis and autophagy in AS, potentially offering new avenues for early diagnosis and therapeutic intervention. Given the intricate interplay between ferroptosis, autophagy, and immune inflammation in AS, we further explored whether these hub genes are associated with immune cell infiltration, thereby linking molecular signatures to the atherosclerotic immune microenvironment.

## 2 MATERIALS AND METHODS

### 2.1 Data acquisition

We screened two datasets related to AS from the GEO public database (https://www.ncbi.nlm.nih.gov/geo/): GSE225650 (GPL24676),^24^ which includes 109 AS samples and 29 normal samples, and GSE163154 (GPL6104),^25^ which was utilized as the validation dataset, containing 16 non‐intraplaque hemorrhage (non‐IPH) samples and 27 intraplaque hemorrhage (IPH) samples. IPH is a recognized hallmark of advanced, unstable atherosclerotic plaques, representing a more severe pathological state compared to non‐IPH lesions. Therefore, this dataset was utilized to validate the diagnostic performance of our identified hub genes in distinguishing between stable and unstable plaques, complementing the primary analysis that compared AS versus normal tissues. To integrate these two datasets generated on different platforms, we employed a two‐step approach. First, cross‐platform merging was performed using the inSilicoMerging R/Bioconductor package.^26^ This package was specifically designed for integrating gene expression data from diverse sources by retaining only common genes across datasets, thereby ensuring comparability.^26^ It provides a unified framework for merging multiple ExpressionSet objects while preserving sample annotations, which is critical for downstream analyses.^26^ Second, to remove residual batch effects introduced by technical variations between the two studies (e.g., different platforms, experimental conditions), we applied the removeBatchEffect function from the limma package.^27^ This method fits a linear model to the data to remove variation attributable to batch effects, while preserving the biological differences of interest by including the original sample groupings as covariates in the model design.^27^ Specifically, we specified the dataset origin (GSE225650 vs. GSE163154) as the batch factor and included the original sample grouping (normal, AS, non‐IPH, and IPH) as covariates in the design matrix to ensure that biologically relevant differences—both between AS and normal tissues and between IPH and non‐IPH lesions—were preserved after batch effect removal. The effectiveness of batch effect removal was evaluated using Uniform Manifold Approximation and Projection (UMAP) dimensionality reduction. As shown in Figure S1A,B, samples clustered primarily by dataset before correction (Figure S1A), whereas after the removeBatchEffect function was applied, samples from both datasets were well integrated (Figure S1B), indicating successful batch effect removal.

**FIGURE 1:**
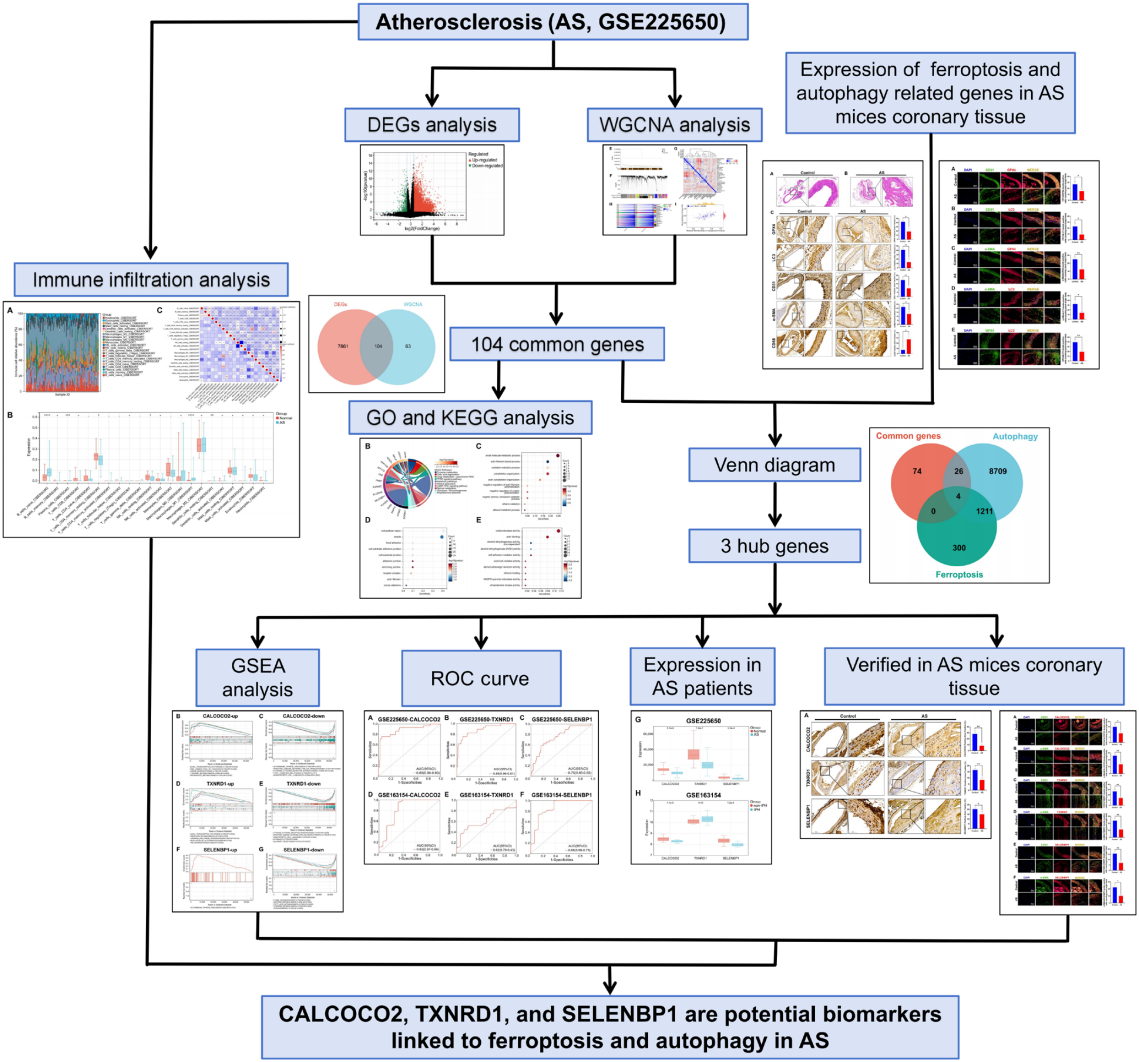
Research flowchart.

The ferroptosis‐related gene set was obtained from the GeneCards database (https://www.genecards.org/, accessed on September 20, 2024), whereas the autophagy‐related datasets were sourced from both the GeneCards database and the Human Autophagy Database (HADb, http://www.autophagy.lu/index.html, accessed on September 20, 2024).^28^, ^29^, ^30^ To ensure comprehensive coverage and avoid prematurely excluding potentially relevant genes, all retrieved genes were included without applying a relevance score threshold. This broad inclusion strategy was subsequently followed by a multistep filtering process—including WGCNA, limma differential expression analysis, ROC curve evaluation, and experimental validation—to ensure the reliability of the final hub genes. The number of genes retrieved at each step was as follows: from GeneCards, we obtained 1515 ferroptosis‐related genes and 9932 autophagy‐related genes; from HADb, we obtained 222 autophagy‐related genes. After duplicates (204 genes overlapped between the two autophagy datasets) were removed, a total of 1515 unique ferroptosis‐related genes and 9950 unique autophagy‐related genes were used for subsequent analysis. The flowchart illustrating this study is presented in Figure 1.

### 2.2 Screening of DEGs

In the GSE225650 dataset, we employed the limma package using R to perform DEG analysis between AS samples and normal samples. The screening criteria were established as adjusted *p* < 0.05 and |log2 fold change(FC)|>0.58.

### 2.3 WGCNA

WGCNA is a systems biology approach that identifies coexpressed gene modules and explores the relationships between key gene modules and various phenotypes.^31^ Initially, we calculated the median absolute deviation (MAD) for each gene and selected the top 50% of genes with the highest MAD values for further analysis. Subsequently, we filtered out outlier genes and samples using the goodSamplesGenes method from the WGCNA package to ensure data quality. Based on this, we constructed a scale‐free coexpression network. The weighted adjacency matrix was generated using the soft‐thresholding parameter “β”, which was then converted into a topological overlap matrix (TOM), while calculating the corresponding dissimilarity (1‐TOM). To classify genes with similar expression profiles into gene modules, we performed average linkage hierarchical clustering based on the TOM dissimilarity measure, setting the minimum module size for the gene dendrogram to 30. Finally, by calculating the dissimilarity of module eigengenes, we determined the cut threshold for the module dendrogram and merged similar modules, ultimately obtaining biologically meaningful gene coexpression modules.

### 2.4 Functional enrichment analysis

GO and KEGG pathway enrichment analyses were performed using the R package clusterProfiler.^32^ For the common genes and the WGCNA‐derived “orangered4” module genes, statistical significance was defined as −log10(*p*‐value) ≥ 1.3 (corresponding to *p* < 0.05). To control for multiple testing, the false discovery rate (FDR) was set at <0.25.

### 2.5 Hub gene identification

To identify hub genes potentially involved in the crosstalk between ferroptosis and autophagy in AS, we employed a sequential overlapping strategy utilizing four gene sets: (1) DEGs associated with atherosclerotic pathogenesis; (2) key module genes derived from WGCNA, which represent functionally coordinated gene networks pertinent to disease phenotypes; (3) ferroptosis‐related genes; and (4) autophagy‐related genes. This multistep intersection was designed to enrich for genes that are not only dysregulated in AS and clustered within coexpression networks but are also annotated to both ferroptosis and autophagy pathways concurrently. In this manner, we prioritized candidate genes with potential roles in the cross‐regulation and crosstalk between ferroptosis and autophagy, rather than focusing on genes involved in either process independently.

### 2.6 GSEA

GSEA analysis was performed using the GSEA package (version 4.4.1) in R. To investigate the potential roles of hub genes, we categorized the samples into high‐expression (≥50%) and low‐expression (<50%) groups based on their expression levels and conducted GSEA for each group. A *p*‐value of <0.05 and an FDR of <0.25 were established as the criteria for significant enrichment.

### 2.7 ROC analysis

We performed an ROC analysis using the R package pROC to obtain the area under the curve (AUC) and the 95% confidence interval.

### 2.8 10‐fold cross‐validation repeated five times

To avoid model overfitting, the diagnostic performance of hub genes was further validated through five repetitions of 10‐fold cross‐validation. Independent validations were conducted on the GSE225650 and GSE163154 datasets, respectively, following this procedure: each dataset was partitioned into 10 mutually exclusive subsets via stratified random sampling, ensuring that each fold maintained a consistent case/control ratio with the original dataset. During each round of cross‐validation, one subset was sequentially used as the test set, whereas the remaining nine subsets were combined to form the training set for model construction and AUC value calculation. Completing 10 iterations, with each subset serving as the test set once, constituted one round of 10‐fold cross‐validation. To mitigate errors arising from random subset partitioning, the aforementioned 10‐fold cross‐validation process was independently repeated five times. Finally, the distribution of AUC values for each repetition of the 10‐fold cross‐validation for each of the three hub genes in the GSE225650 and GSE163154 datasets, respectively, was visualized using boxplots, intuitively illustrating the stability and fluctuation range of their diagnostic performance.

### 2.9 Immune infiltration and immune correlation analysis of hub genes

CIBERSORT is a method for analyzing the cellular composition of complex tissues based on gene expression profiles, which can be utilized to evaluate the proportion of immune cells in tissue samples.^33^ Immune infiltration analysis was performed on the GSE225650 dataset (original, non‐merged coronary artery samples) using the CIBERSORT algorithm with the default LM22 signature matrix, which defines 22 immune cell subtypes. Relative proportions of these immune cells were estimated for each sample^34^, and samples with CIBERSORT output *p* < 0.05 were retained for downstream analysis.^33^

The bar chart provides an intuitive representation of the percentages of 22 immune cells in each sample across different groups. Furthermore, the R package vioplot can be employed to visualize the differences in immune cell expression between the AS group and the normal group, while the corrplot package can be used to analyze the correlations among immune cells.^35^

It is important to acknowledge the inherent limitations of deconvolution approaches when applied to vascular tissues, such as the coronary artery.^34^ Unlike blood, arterial tissue possesses a unique and complex cellular composition, which includes endothelial cells, VSMCs, fibroblasts, and adipocytes, in addition to infiltrating immune cells. The LM22 signature matrix was primarily derived from purified hematopoietic cell populations; therefore, the estimated immune cell proportions represent relative abundances within the immune compartment rather than absolute percentages of the total tissue cellularity.^36^ Furthermore, the presence of non‐immune cell types may influence the accuracy of deconvolution.^34^ Despite these limitations, CIBERSORT remains a widely used and validated tool for comparing immune infiltration patterns between disease and control groups in solid tissues.^36^

To further explore the immune modulatory roles of the identified hub genes (CALCOCO2, TXNRD1, and SELENBP1), Spearman's correlation analysis was performed between their expression levels and the relative abundance of the 22 immune cell types estimated using CIBERSORT. Correlation coefficients (*r*) and statistical significance (−log10 transformed *p*‐values) were calculated and visualized as a heatmap using the R package pheatmap. Statistical significance was defined as −log10(*p*) ≥ 1.3, corresponding to *p* ≤ 0.05.

### 2.10 AS mouse model

Male *Apoe* ^−/−^ mice (C57BL/6J background), aged 8 weeks and weighing 20–22 g at the start of the experiment, were used in this study. *Apoe* ^−/−^ mice were divided into two groups: the control group (Control, *n* = 3), which was fed a standard diet, and the atherosclerosis model group (AS, *n* = 3), which was fed a high‐cholesterol, high‐fat diet (HCFD) composed of 81.5% maintenance chow, 10% lard, 5% sucrose, and 3.5% cholesterol for 10 consecutive months to induce atherosclerotic lesions. All mice were housed under specific pathogen‐free conditions with a 12‐h light/dark cycle. Animal protocols were approved by the Institutional Animal Care and Use Committee (IACUC) of Chinese PLA General Hospital (approval ID: 2023‐X19‐168).

### 2.11 Hematoxylin–eosin staining

The paraffin blocks of mouse coronary artery tissue were sectioned into slices of 3 μm thickness using a paraffin microtome. These slices were then baked on a slide warmer at 55°C for 2 h, followed by dewaxing and rehydration using xylene and graded alcohols. The nuclei were stained with hematoxylin, whereas the cytoplasm was stained with eosin. Subsequently, the slices were dehydrated through graded alcohols, cleared in xylene, and mounted with neutral balsam. Finally, the slices were scanned using the SOS‐40P digital pathology slide scanner.

### 2.12 Immunohistochemistry staining

Mouse coronary artery tissue slices were baked on a slide warmer at 55°C for 2 h, followed by dewaxing and rehydration using xylene and graded alcohols. High‐pressure antigen retrieval was performed in a pH 6.0 citrate buffer for 5 min. After cooling to room temperature, the slices were washed thrice with phosphate‐buffered saline (PBS) buffer, then incubated in a 3% hydrogen peroxide solution for 10 min to eliminate endogenous peroxidase activity. Finally, the slices were blocked with 10% goat serum for 2 h. The primary antibody was applied and incubated overnight at 4°C. The primary antibodies used included GPX4 (DF6701), LC3 (AF5402), CD31 (66065‐2‐Ig), α‐SMA (ab7817), CD68 (DF7518), CALCOCO2 (DF12360), TXNRD1 (bs‐8299R), and SELENBP1 (bs‐4200R). After being washed with PBS, the sections were treated with a secondary antibody corresponding to the species of the primary antibody and incubated at room temperature for 1 h. A freshly prepared 3,3′‐diaminobenzidine (DAB) chromogen solution was applied, with the chromogenic time controlled under the microscope, and the chromogenic reaction was terminated with ddH_2_O, followed by nuclear staining with hematoxylin. Finally, the slides were dehydrated, cleared, and mounted. The slides were scanned using the SOS‐40P digital pathology slide scanner.

### 2.13 IF staining

The procedural steps before the addition of the primary antibody are consistent with the initial stages of IHC staining. After this, the sections were incubated overnight at 4°C with the following combinations of antibodies: mouse anti‐CD31 and rabbit anti‐GPX4 antibodies, mouse anti‐CD31 and rabbit anti‐LC3 antibodies, mouse anti‐α‐SMA and rabbit anti‐GPX4 antibodies, mouse anti‐α‐SMA and rabbit anti‐LC3 antibodies, mouse anti‐GPX4 (67763‐1‐Ig) and rabbit anti‐LC3 antibodies, mouse anti‐CD31 and rabbit anti‐CALCOCO2 antibodies, mouse anti‐α‐SMA and rabbit anti‐CALCOCO2 antibodies, mouse anti‐CD31 and rabbit anti‐TXNRD1 antibodies, mouse anti‐α‐SMA and rabbit anti‐TXNRD1 antibodies, mouse anti‐CD31 and rabbit anti‐SELENBP1 antibodies, and mouse anti‐α‐SMA and rabbit anti‐SELENBP1 antibodies. After being washed with PBS, a mixture of goat anti‐rabbit (ab150079) and goat anti‐mouse (ab150113) secondary antibodies (1:500) was applied to the sections, followed by incubation at room temperature in the dark for 1 h. After three washes with PBS, the sections were mounted using a mounting medium containing DAPI. Fluorescence signals were subsequently observed under a fluorescence microscope (Olympus BX53).

### 2.14 Statistical analysis

The statistical analysis for this study was primarily performed using R software. All IHC and IF experiments were performed a minimum of three times. The fraction of positive area (%) in IHC experiments was calculated using ImageJ software. For the counting of double‐positive cells in IF costaining, a semiquantitative counting method was adopted, which was combined with objective quantification using ImageJ software for all IF staining samples. Specifically, three independent researchers, who were blinded to group allocation, performed the semiquantitative counting. Concurrently, ImageJ software was used to objectively count the number of double‐positive cells, with the average value from both methods serving as the final result. Data analysis and graph generation were accomplished using GraphPad Prism software (version 9.0). Statistical analysis was performed using an unpaired Student's *t*‐test. The threshold for statistical significance was established at *p* < 0.05.

## 3 RESULTS

### 3.1 The identification of DEGs and key coexpression modules in AS and normal patients

**FIGURE 2:**
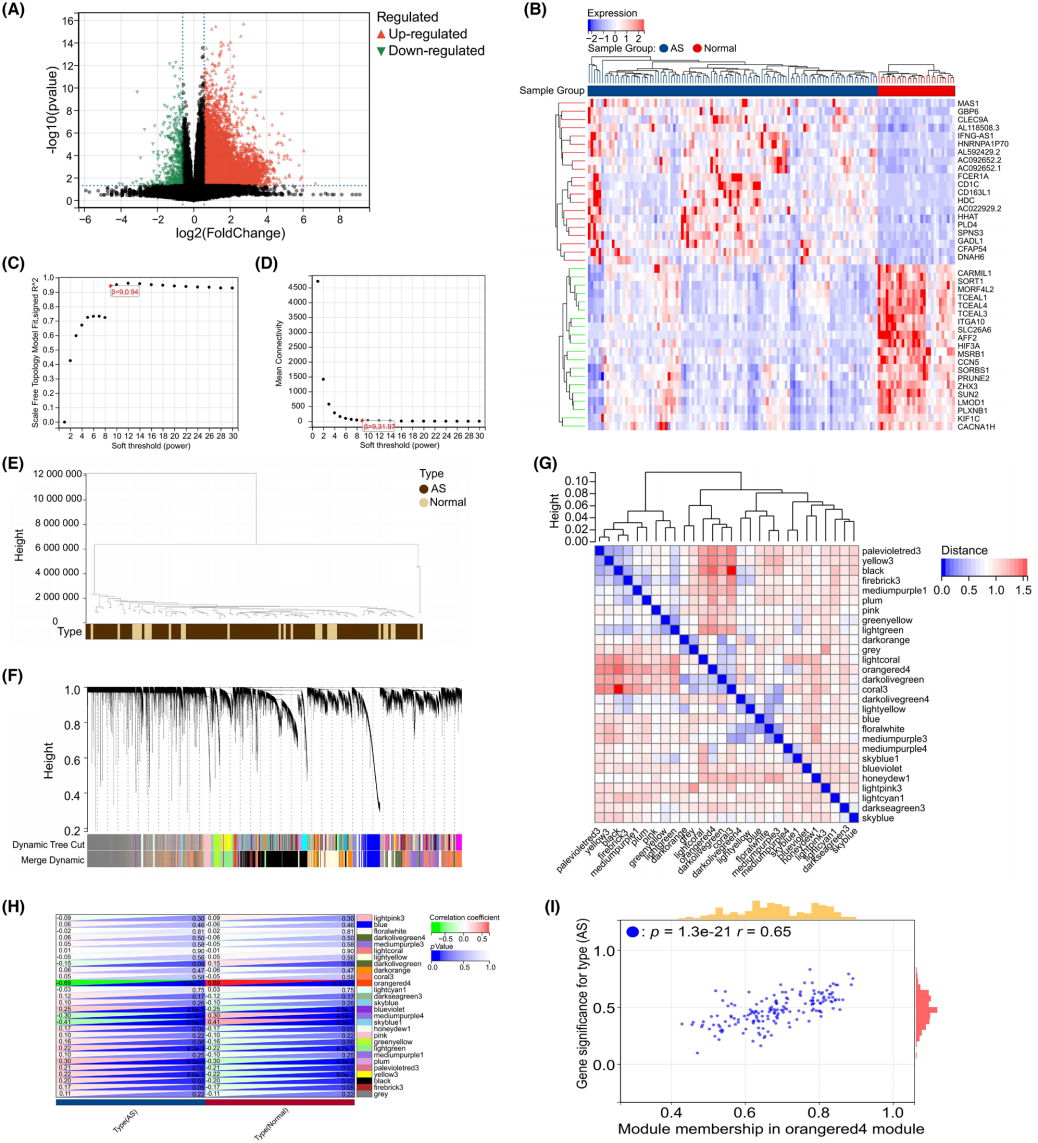
The identification of differentially expressed genes (DEGs) and key coexpression modules in atherosclerosis (AS) and normal patients. (A) Volcano plot illustrates DEGs between AS and normal patients. Red points denote upregulated genes in AS, green points denote downregulated genes in the AS, and black points represent nonsignificant expression differences. (B) Heatmap displaying the expression patterns of the top 20 upregulated and top 20 downregulated DEGs between AS and normal samples. (C, D) Weighted Gene Coexpression Network Analysis (WGCNA): Selection of the soft‐thresholding power (*β*). (C) Analysis of the scale‐free fit index. (D) Analysis of the mean connectivity. A *β* value of 9 (scale‐free *R* ^2^ = 0.94) was chosen. (E) Gene clustering dendrogram based on WGCNA. (F) Identified gene coexpression modules. Different colors represent distinct modules. (G) Heatmap of eigengene adjacency matrix. (H) Heatmap showing the relationships between different modules and trait. (I) Scatter plot illustrates the correlation between module membership in the orangered4 module and gene significance in AS.

The AS dataset GSE225650, comprising 29 normal samples and 109 AS samples, was obtained from the GEO database. Differential expression analysis using limma package identified 7965 DEGs between AS and normal samples. Of these, 7454 were upregulated and 511 were downregulated in AS (Figure 2A,B). Subsequently, WGCNA was performed to identify gene modules associated with AS. A soft‐thresholding power of *β* = 9 (scale‐free *R* ^2^ = 0.94) was selected based on criteria of scale independence and mean connectivity (Figure 2C,D). This threshold was used to construct a gene coexpression network, from which 28 distinct coexpression modules were identified through hierarchical clustering (Figure 2E–G).

The orangered4 module, containing 167 genes, exhibited the strongest correlation with AS status (*r* = −0.69, *p* = 4.4e−21; Figure 2H). A significant positive correlation was observed between module membership within the orangered4 module and the gene significance for AS (*r* = 0.65, *p* = 1.3e−21; Figure 2I), further underscoring this module's strong association with AS.

To evaluate the biological relevance of the orangered4 module, we performed GO and KEGG pathway analyses on the 167 genes within this module. As summarized in Figure S2, the module demonstrated significant enrichment in pathways closely related to AS pathogenesis, including small molecule metabolic process, oxidation–reduction process, lipid metabolic process, positive regulation of signal transduction, and actin filament‐based process (biological process); cytosol, extracellular region, and vesicle (cellular component); and oxidoreductase activity, actin binding, and phosphatidylinositol‐4,5‐bisphosphate binding (molecular function). KEGG pathway analysis revealed significant enrichment in metabolic pathways, fatty acid degradation, Rap1 signaling pathway, cGMP‐PKG signaling pathway, and regulation of actin cytoskeleton (Figure S2). Notably, Rap1 signaling has been recently implicated in endothelial ferroptosis and AS progression via the Rap1B/NRF2/GPX4 axis.^37^ These enrichment results confirm that the orangered4 module is not only statistically associated with AS but also biologically engaged in core pathological mechanisms relevant to our study focus—ferroptosis–autophagy crosstalk and immune modulation.

In addition to the orangered4 module, several other modules showed significant correlations with AS, including the skyblue1 (*r* = −0.41, *p* = 6.5e−7), plum (*r* = 0.30, *p* = 4.3e−4), and mediumpurple4 (*r* = −0.30, *p* = 4.1e−4) modules (Figure 2H). Detailed functional characterization of these modules was beyond the scope of the current study, as our primary aim was to identify the module most strongly associated with AS for subsequent hub gene identification. The orangered4 module was selected based on two criteria: (i) it exhibited the highest correlation coefficient among all modules; and (ii) its functional enrichment profile most closely aligned with our research focus on ferroptosis–autophagy crosstalk and immune modulation.

Therefore, the orangered4 module was selected as the key module for subsequent analyses.

### 3.2 Functional enrichment analysis of common genes

**FIGURE 3:**
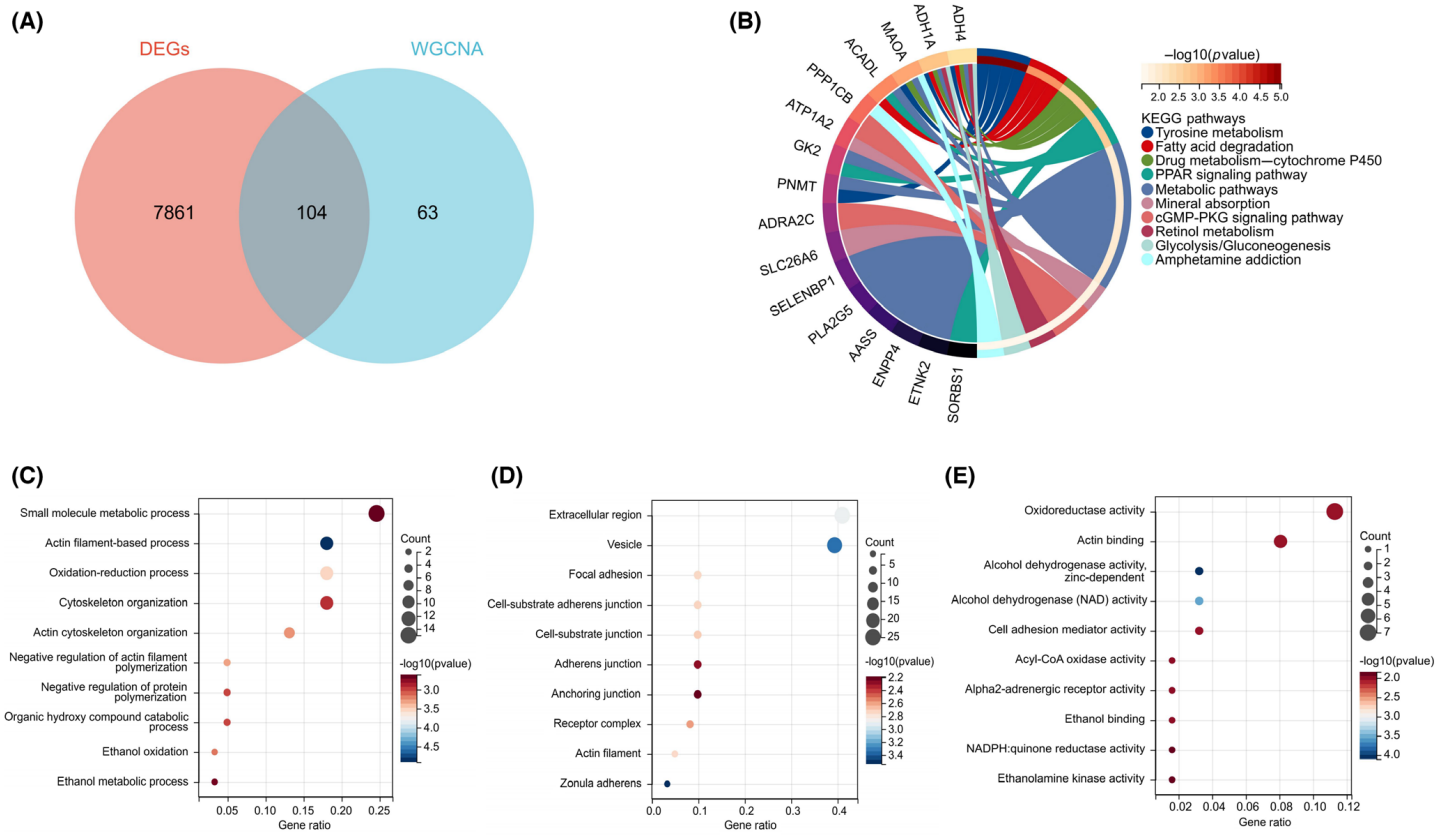
Functional enrichment analysis of common genes. (A) Venn diagram showing the intersection of differentially expressed genes (DEGs) and genes from the orangered4 module, yielding 104 common genes. (B) Kyoto Encyclopedia of Genes and Genomes (KEGG) pathway analysis of these common genes. (C–E) Gene Ontology (GO) enrichment analysis of the common genes. Dot plots show top enriched GO terms in (C) biological process, (D) cellular component, and (E) molecular function categories.

To identify genes robustly associated with AS, we intersected the previously identified DEGs with genes from the key orangered4 module. This intersection yielded 104 common genes (Figure 3A), which were then subjected to GO and KEGG pathway analyses to elucidate their biological functions.

KEGG pathway analysis indicated that these common genes are primarily enriched in metabolic pathways, fatty acid degradation, and tyrosine metabolism (Figure 3B). GO analysis revealed significant enrichment in several categories. For biological processes, top enriched terms included actin filament‐based process, oxidation–reduction process, and small molecule metabolic process. In terms of cellular components, common genes were mainly enriched in vesicle, extracellular region, and focal adhesion. Regarding molecular functions, predominant terms were alcohol dehydrogenase activity (zinc‐dependent), oxidoreductase activity, and actin binding (Figure 3C–E).

### 3.3 Ferroptosis activation and autophagy inhibition in AS mice model

**FIGURE 4:**
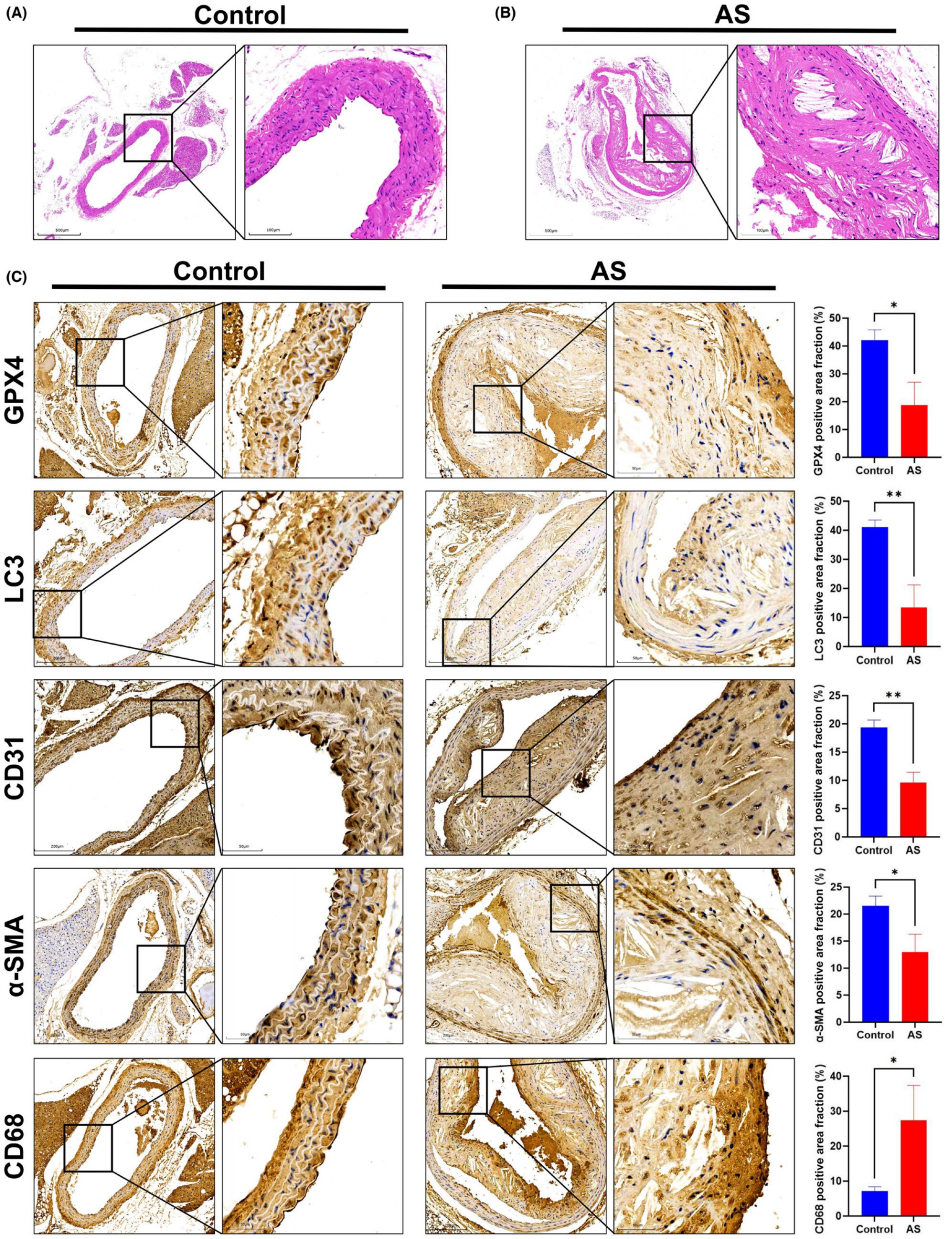
Altered expression of ferroptosis and autophagy markers in coronary tissue of atherosclerosis (AS) mice. (A, B) Hematoxylin–eosin (HE) staining of coronary tissue from (A) control mice and (B) AS mice. (C) Immunohistochemistry (IHC) staining and quantification (positive area fraction, %) of GPX4 (ferroptosis marker), LC3 (autophagy marker), CD31 (endothelial cell marker), α‐SMA (vascular smooth muscle cell marker), and CD68 (macrophage marker) in coronary artery tissues from control and AS mice. Data are presented as mean ± standard deviation (SD). *p < 0.05, **p < 0.01 versus control.

To investigate AS pathogenesis in vivo, coronary artery tissues were collected from *Apoe* ^−/−^ mice fed a high‐fat diet for 10 months and processed for paraffin embedding. HE staining was then performed to assess morphological changes. Compared to control mice, the coronary artery lumen in AS mice was significantly narrowed, exhibited typical atherosclerotic plaque characteristics, including intimal thickening, necrotic core formation, and fibrous cap development, confirming the successful establishment of the AS mouse model (Figure 4A,B). IHC analysis was used to assess markers of ferroptosis and autophagy. GPX4, a key negative regulator of ferroptosis that eliminates LPO, showed significantly diminished protein expression in atherosclerotic lesions, indicative of ferroptosis activation (Figure 4C). Conversely, protein levels of LC3, a marker for autophagosomes, where conversion from LC3I to LC3II signals autophagy activation, were decreased in these lesions, suggesting autophagy inhibition (Figure 4C). Given that endothelial cells, VSMCs, and macrophages are principal components of atherosclerotic plaques, their status was also examined. IHC analyses showed significantly downregulated CD31, an endothelial marker, expression in the AS group compared to controls (Figure 4C). In contrast, the expression of α‐SMA, a VSMC marker, increased in the intimal region but decreased in the medial region within the AS group (Figure 4C). Furthermore, CD68, a macrophage marker, expression was significantly elevated in the intimal region of AS plaques (Figure 4C).

**FIGURE 5:**
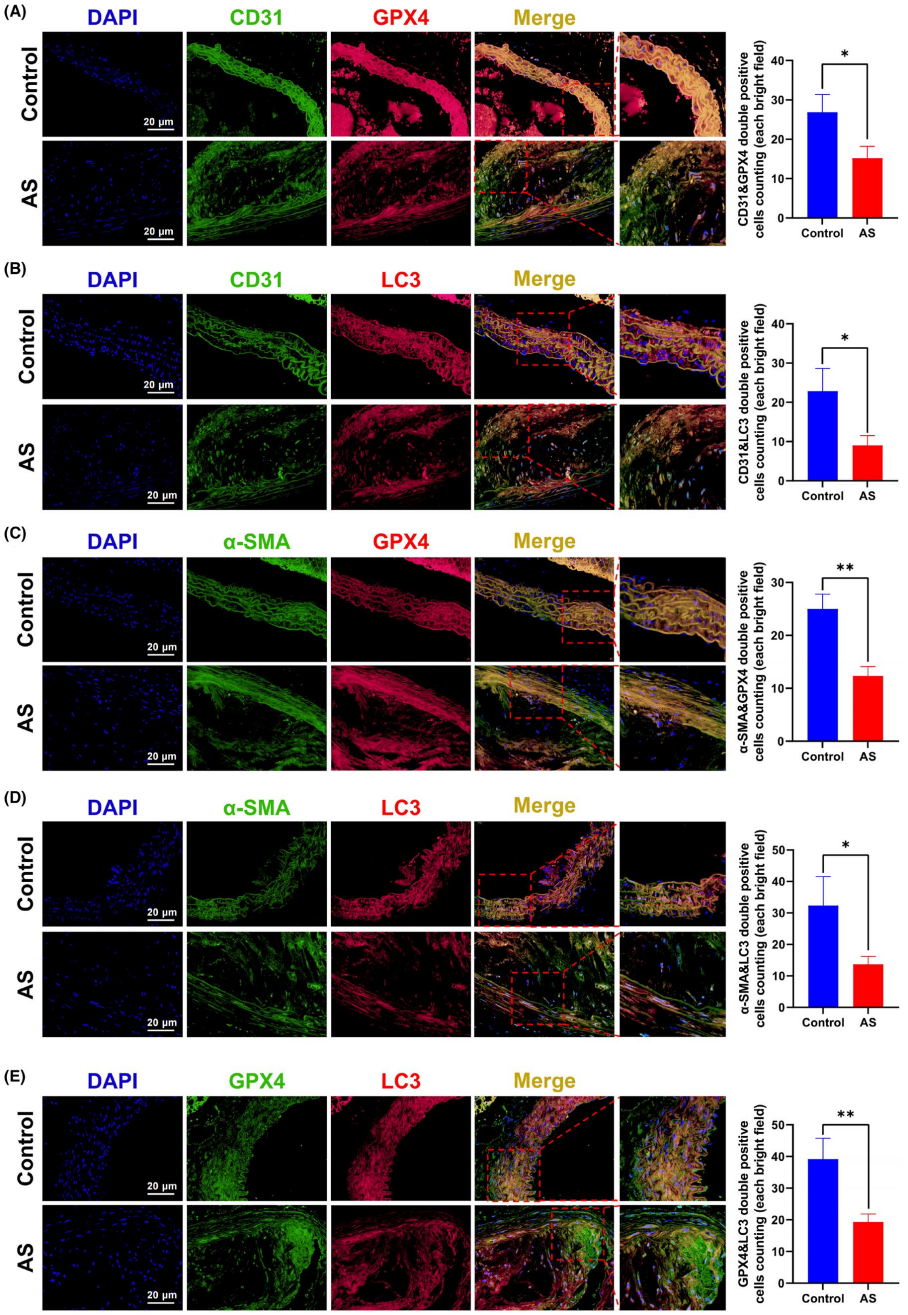
Cellular localization and expression of ferroptosis and autophagy markers in coronary tissues of atherosclerosis (AS) mice. (A–E) Immunofluorescence (IF) colocalization staining and double positive cells counting (each bright field) for CD31&GPX4, CD31&LC3, α‐SMA&GPX4, α‐SMA&LC3, and GPX4&LC3 in the coronary tissue of both control and AS mice. Data are presented as mean ± standard deviation (SD). *p < 0.05, **p < 0.01.

IF colocalization studies further explored these processes in specific cell types. GPX4 and LC3 proteins partially colocalized with CD31, suggesting that ferroptosis and autophagy occur in endothelial cells (Figure 5A,B). The number of CD31&GPX4 and CD31&LC3 double‐positive cells was significantly lower in the AS group compared to the control group (Figure 5A,B). Similarly, GPX4 and LC3 partially colocalized with α‐SMA, indicating that these processes also occur in VSMCs (Figure 5C,D). α‐SMA&GPX4 and α‐SMA&LC3 double‐positive cells were significantly reduced in AS mice (Figure 5C,D). Notably, overlapping fluorescence for GPX4 and LC3 suggested a potential interplay between ferroptosis and autophagy, with the number of GPX4&LC3 double‐positive cells also significantly decreased in the AS group (Figure 5E). Collectively, these findings indicate that in the coronary artery tissues of this AS mouse model, ferroptosis is activated while autophagy is suppressed, particularly within endothelial cells and VSMCs.

### 3.4 Identification and GSEA of hub genes linked to ferroptosis and autophagy in AS

**FIGURE 6:**
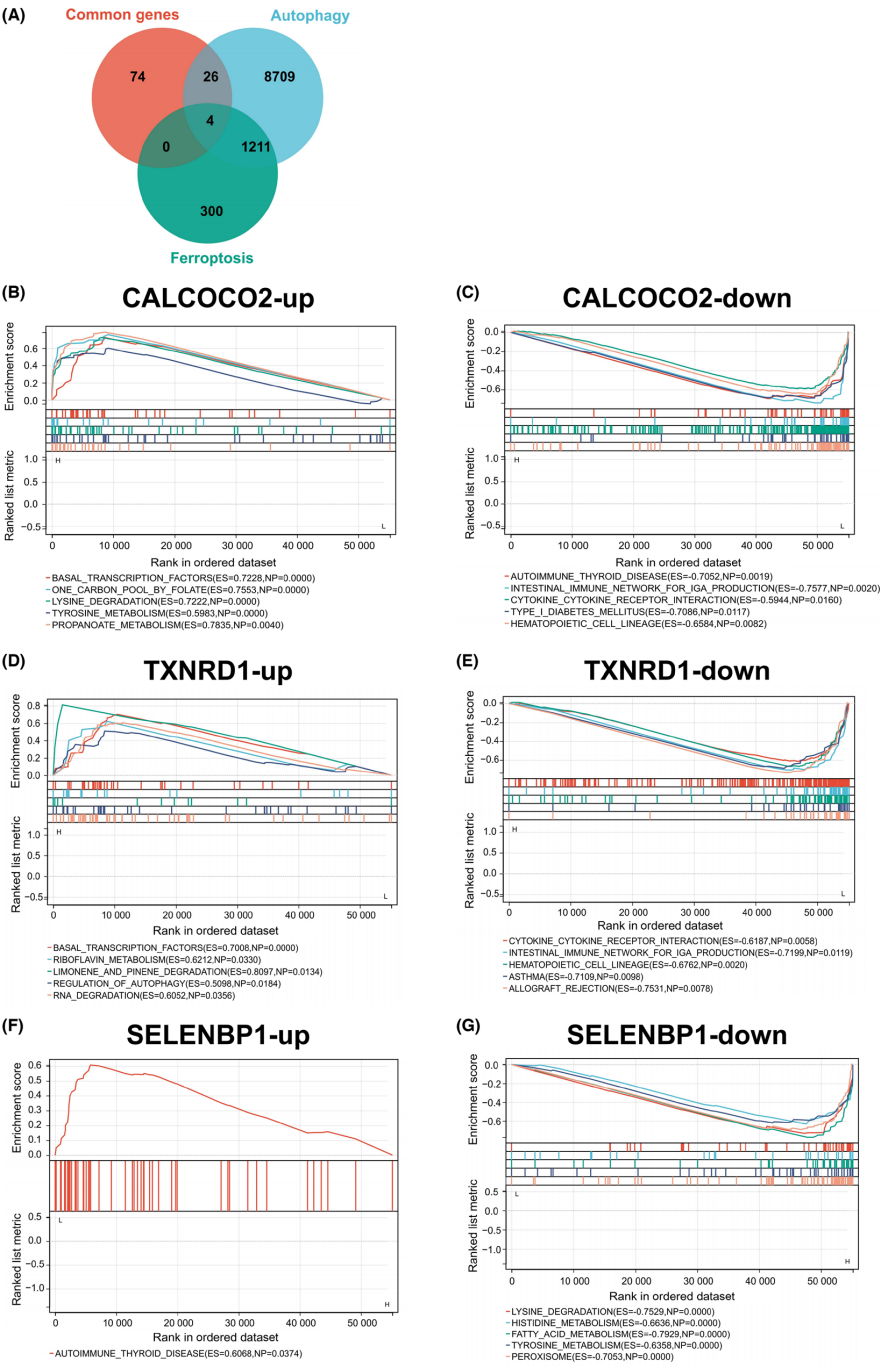
Identification and Gene Set Enrichment Analysis (GSEA) of hub genes linked to ferroptosis and autophagy in atherosclerosis (AS). (A) Venn diagram showing the intersection of the 104 common genes, ferroptosis‐related genes (*n* = 1515), and autophagy‐related genes (*n* = 9950). Four genes—CALCOCO2, TXNRD1, SELENBP1, and LINC00844—were commonly identified across all three gene sets. LINC00844, a long noncoding RNA, was excluded from subsequent protein‐level analyses due to the study's focus on protein‐coding genes. (B, C) GSEA analysis of CALCOCO2‐up and CALCOCO2‐down. (D, E) GSEA analysis of TXNRD1‐up and TXNRD1‐down. (F, G) GSEA analysis of SELENBP1‐up and SELENBP1‐down.

To identify hub genes potentially governing the crosstalk between ferroptosis and autophagy in AS, we conducted an integrative analysis of three gene sets: (i) the 104 common genes previously identified by intersecting DEGs and WGCNA module genes, (ii) ferroptosis‐related genes (*n* = 1515 from GeneCards), and (iii) autophagy‐related genes (*n* = 9950 from GeneCards and HADb). The intersection of these three sets revealed four potential hub genes: CALCOCO2, TXNRD1, and SELENBP1 (protein‐coding genes), along with LINC00844 (a long intergenic noncoding RNA) (Figure 6A). LINC00844 was excluded from subsequent protein‐focused analyses because our downstream validation experiments—including IHC and IF—were designed specifically for protein‐coding genes. Nevertheless, its identification suggests that noncoding RNAs may also contribute to ferroptosis–autophagy crosstalk in AS, warranting independent investigation in future studies. GSEA was then performed on the remaining three protein‐coding hub genes.

GSEA revealed distinct pathway enrichments for each hub gene. For CALCOCO2, samples with high expression were significantly enriched in pathways, including basal transcription factors, one‐carbon pool by folate, lysine degradation, tyrosine metabolism, and propanoate metabolism (Figure 6B). Conversely, low CALCOCO2 expression was associated with enrichment in autoimmune thyroid disease, intestinal immune network for immunoglobulin A (IgA) production, cytokine–cytokine receptor interaction, type 1 diabetes mellitus, and hematopoietic cell lineage (Figure 6C).

For TXNRD1, high‐expression samples showed significant enrichment in basal transcription factors, riboflavin metabolism, limonene and pinene degradation, regulation of autophagy, and RNA degradation (Figure 6D). In contrast, low TXNRD1 expression was linked to enrichment in cytokine–cytokine receptor interaction, intestinal immune network for IgA production, hematopoietic cell lineage, asthma, and allograft rejection (Figure 6E).

Finally, for SELENBP1, high expression was primarily associated with enrichment in autoimmune thyroid disease pathways (Figure 6F), whereas low expression correlated with significant enrichment in lysine degradation, histidine metabolism, fatty acid metabolism, tyrosine metabolism, and peroxisome (Figure 6G).

### 3.5 Diagnostic value assessment of hub genes and its expression in AS patients

**FIGURE 7:**
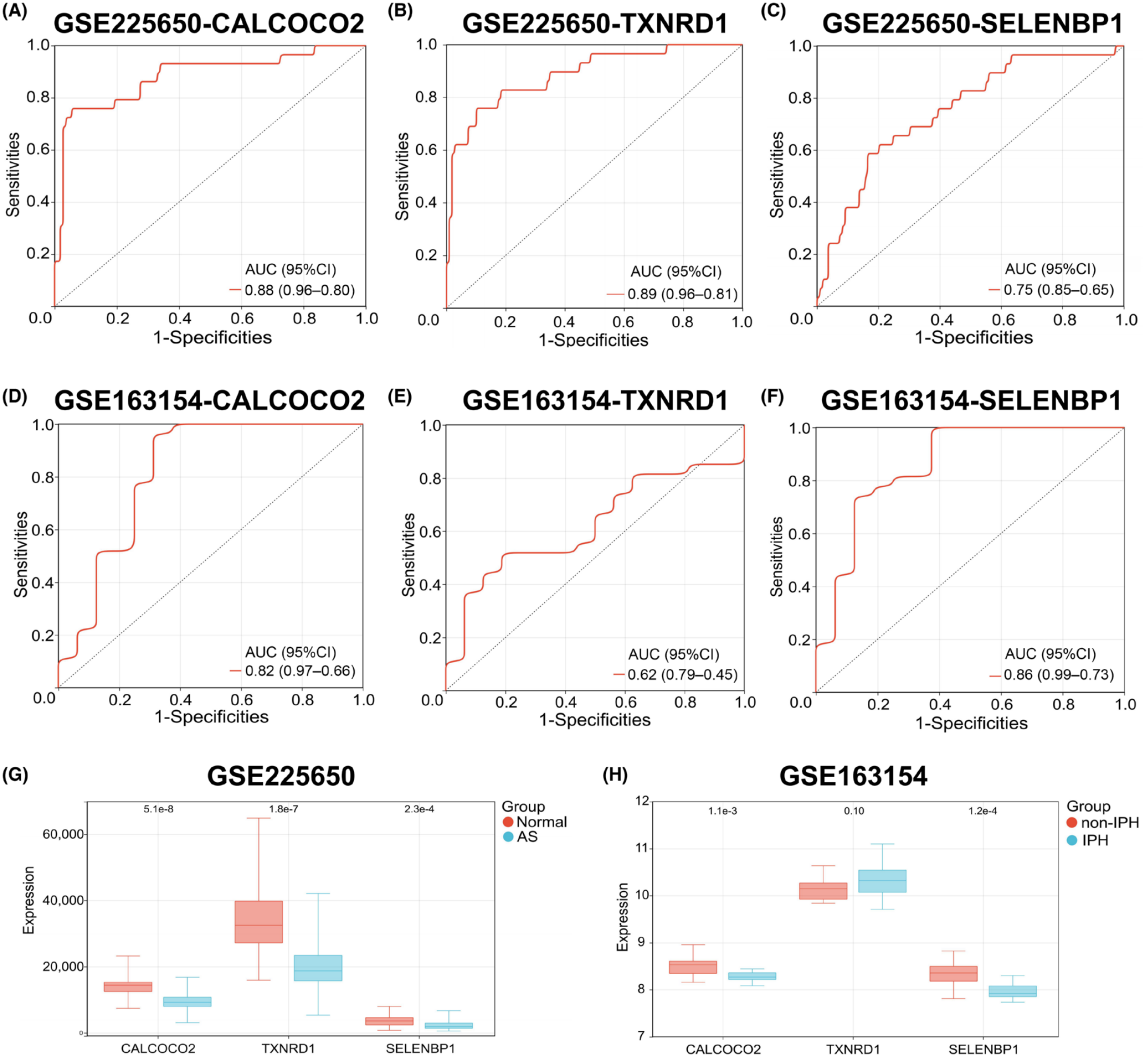
Evaluation of diagnostic value and expression levels of hub genes in atherosclerosis (AS) patients. (A–C) Receiver operating characteristic (ROC) curve analysis of hub genes in the GSE225650 dataset: (A) CALCOCO2 (AUC = 0.88), (B) TXNRD1 (AUC = 0.89), (C) SELENBP1 (AUC = 0.75). (D–F) ROC curve analysis of hub genes in the GSE163154 validation dataset: (D) CALCOCO2 (AUC = 0.82), (E) TXNRD1 (AUC = 0.62), (F) SELENBP1 (AUC = 0.86). (G) Relative expression levels of CALCOCO2, TXNRD1, and SELENBP1 in AS versus normal samples from the GSE225650 dataset. The *Y*‐axis represents normalized expression values. (H) Relative expression levels of CALCOCO2, TXNRD1, and SELENBP1 in different sample groups from the GSE163154 dataset. The *Y*‐axis represents normalized expression values.

To evaluate the diagnostic efficacy of the three hub genes, ROC analysis and 10‐fold cross‐validation repeated five times were performed. In the GSE225650 dataset, CALCOCO2 (AUC = 0.88), TXNRD1 (AUC = 0.89), and SELENBP1 (AUC = 0.75) demonstrated robust diagnostic performance (Figure 7A–C). These findings were further assessed in the external dataset GSE163154, which yielded AUC values of 0.82 for CALCOCO2, 0.62 for TXNRD1, and 0.86 for SELENBP1 (Figure 7D–F).

Notably, although CALCOCO2 and SELENBP1 maintained consistent diagnostic performance across both datasets, TXNRD1 exhibited marked instability, with its AUC dropping from 0.89 to 0.62. The 10‐fold cross‐validation boxplots confirmed this finding: CALCOCO2 and SELENBP1 showed stable AUC distributions across iterations in both datasets, whereas TXNRD1 displayed wide interquartile ranges and outlier values in the validation dataset (GSE163154), indicating greater variability in diagnostic performance (Figure S3A–F). These results indicate that TXNRD1 is not reliable as a stand‐alone diagnostic biomarker for AS.

Analysis of hub gene expression in these datasets revealed that CALCOCO2 and SELENBP1 were significantly downregulated in patients with AS across both datasets (Figure 7G,H). TXNRD1 was also significantly downregulated in AS patients in the GSE225650 dataset. However, in the GSE163154 dataset, although TXNRD1 expression showed an upward trend in AS, this difference was not statistically significant (Figure 7G,H).

### 3.6 Immune infiltration landscape and its correlation with hub genes

**FIGURE 8:**
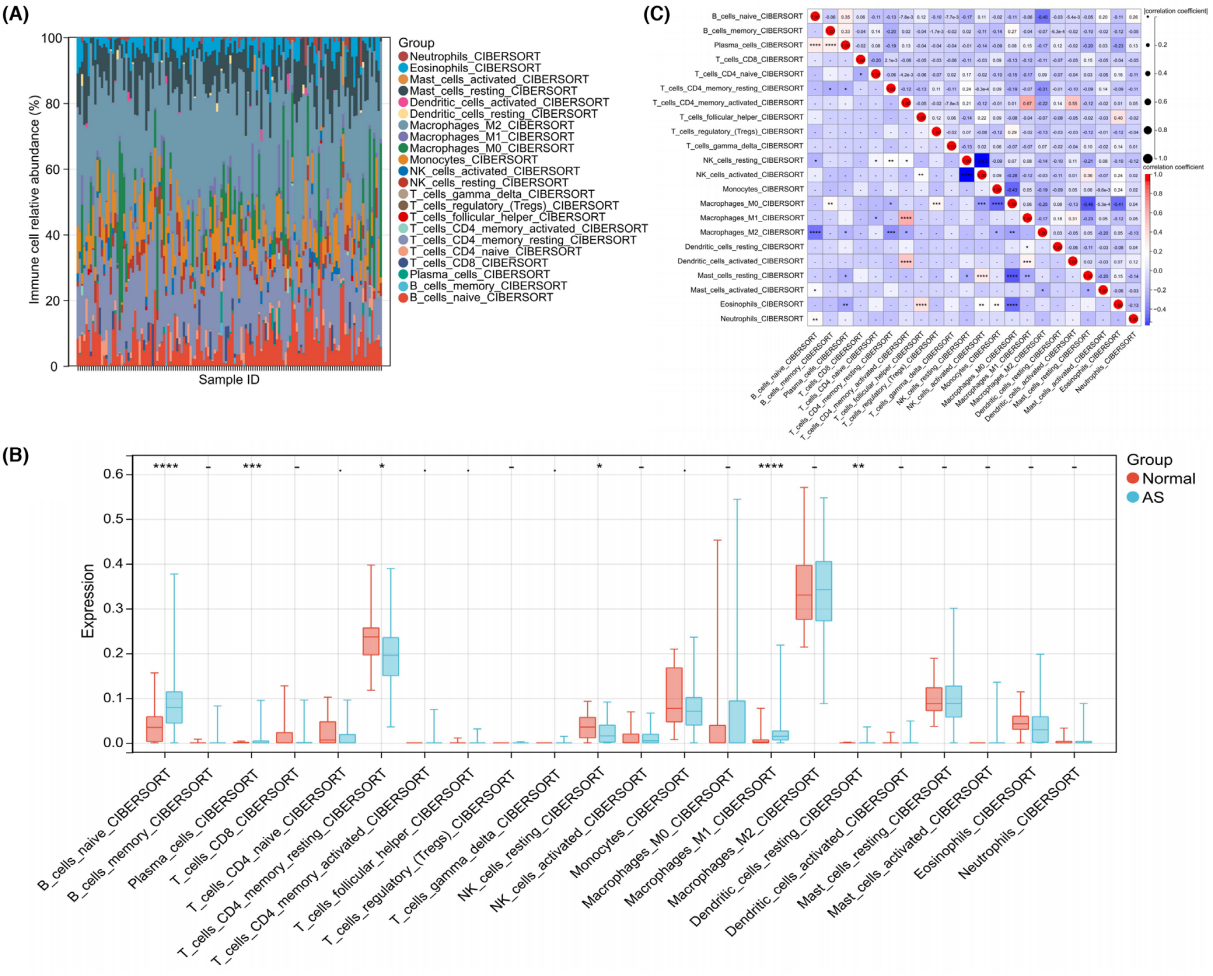
Immune infiltration analysis. (A) The relative abundance of 22 immune cell types in each sample from the normal and atherosclerosis (AS) groups. (B) The differences in the expression levels of various immune cells between the normal group and the AS group. (C) Correlation analysis of the 22 immune cells. *p < 0.05, **p < 0.01, ***p < 0.001, ****p < 0.0001.

To evaluate the role of immune regulation in AS, the immune cell infiltration landscape was analyzed. The percentages of 22 distinct immune cell types within each sample from both the normal and AS groups are illustrated in Figure 8A. Compared to the normal group, patients with AS exhibited elevated levels of naive B cells, plasma cells, M1 macrophages, and resting dendritic cells, alongside reduced levels of resting CD4 memory T cells and resting NK cells (Figure 8B). Correlation analysis among the 22 immune cell types revealed that activated CD4 memory T cells were significantly positively correlated with M1 macrophages (*r* = 0.67) and activated dendritic cells (*r* = 0.55) (Figure 8C). Conversely, resting NK cells showed a significant negative correlation with activated NK cells (*r* = −0.53) (Figure 8C).

To further investigate the association between the identified hub genes and the observed immune alterations, we conducted a Spearman correlation analysis between the expression levels of CALCOCO2, TXNRD1, and SELENBP1 and the relative abundance of 22 immune cell types in the GSE225650 dataset. As illustrated in Figure S4, CALCOCO2 expression exhibited a significant negative correlation with M1 macrophages (*r* = −0.37, *p* < 0.0001) and M0 macrophages (*r* = −0.31, *p* < 0.001), while displaying a positive correlation with resting memory CD4 T cells (*r* = 0.26, *p* < 0.01), monocytes (*r* = 0.28, *p* < 0.001), and eosinophils (*r* = 0.24, *p* < 0.01). TXNRD1 revealed strong negative correlations with M1 macrophages (*r* = −0.36, *p* < 0.0001) and M0 macrophages (*r* = −0.24, *p* < 0.01), as well as with naive B cells (*r* = −0.29, *p* < 0.001) and plasma cells (*r* = −0.25, *p* < 0.01); conversely, it was positively correlated with resting memory CD4 T cells (*r* = 0.27, *p* < 0.01), resting mast cells (*r* = 0.24, *p* < 0.01), and eosinophils (*r* = 0.25, *p* < 0.01) (Figure S4). SELENBP1 was significantly negatively correlated with M1 macrophages (*r* = −0.27, *p* < 0.01), M0 macrophages (*r* = −0.23, *p* < 0.01), and activated memory CD4 T cells (*r* = −0.18, *p* < 0.05), while showing positive correlations with resting mast cells (*r* = 0.21, *p* < 0.05) and eosinophils (*r* = 0.18, *p* < 0.05) (Figure S4). These findings indicate that all three hub genes are significantly associated with various immune cell populations, particularly those exhibiting altered infiltration in AS, thereby supporting their potential roles as immune modulators in the progression of AS.

In summary, AS patients demonstrate notable alterations in the infiltration of multiple immune cell types, suggesting that these cells could be potential therapeutic targets. Furthermore, the strong correlations between the hub genes and key immune populations provide a direct link between the molecular signatures identified in this study and the immune microenvironment of atherosclerotic plaques.

### 3.7 Expression validation of hub genes in AS mice model

**FIGURE 9:**
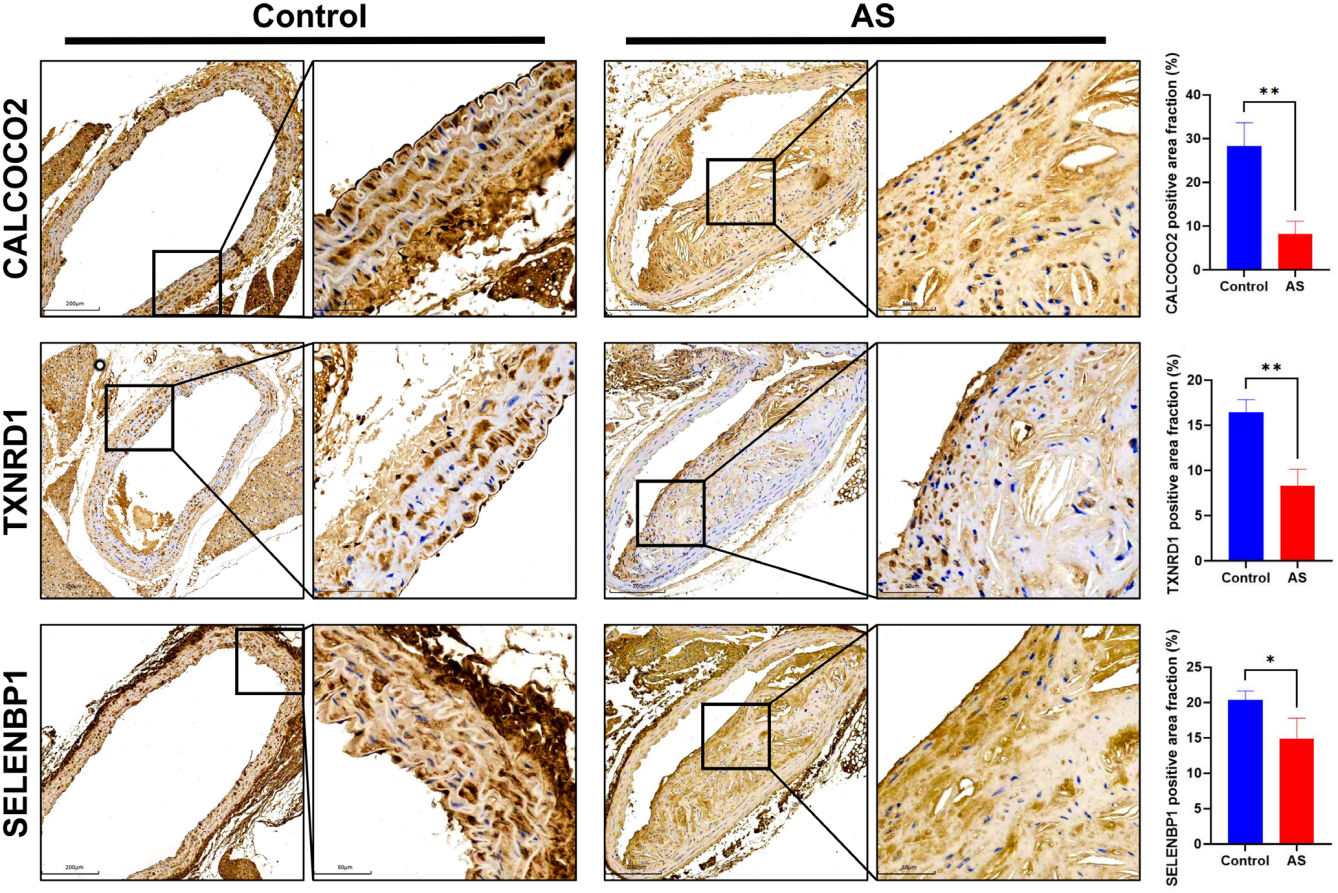
Expression levels of hub genes in the coronary tissue of control and atherosclerosis (AS) mice. (A) Immunohistochemistry (IHC) staining and the positive area fraction (%) of CALCOCO2, TXNRD1, and SELENBP1 in the coronary tissue of both control and AS mice. Data are presented as mean ± standard deviation (SD). *p < 0.05, **p < 0.01 versus control.

To further substantiate the relevance of the identified hub genes to AS, the expression levels were assessed in coronary artery tissues from an AS mouse model. IHC analyses revealed that protein expression of CALCOCO2, TXNRD1, and SELENBP1 was significantly diminished in the coronary artery tissues of AS mice compared to controls (Figure 9).

**FIGURE 10:**
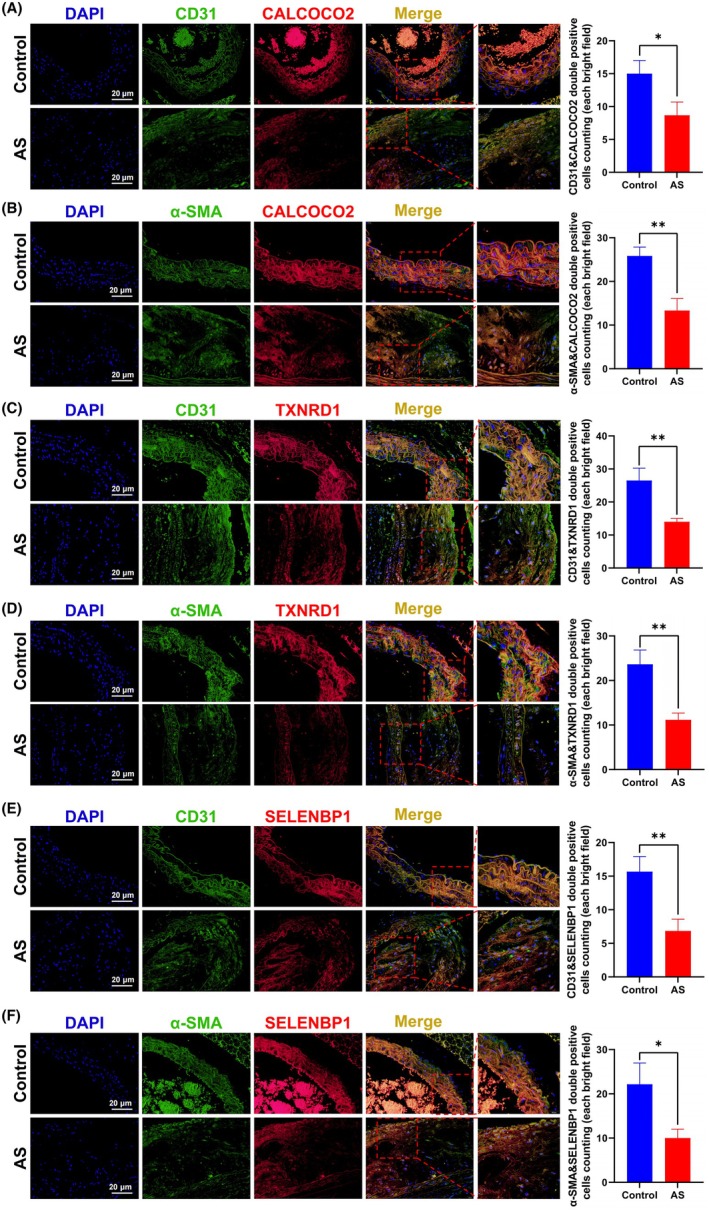
Cellular expression of hub genes in coronary tissues of atherosclerosis (AS) mice using immunofluorescence (IF). (A–F) IF colocalization staining and double positive cells counting for CD31&CALCOCO2, α‐SMA&CALCOCO2, CD31&TXNRD1, α‐SMA&TXNRD1, CD31&SELENBP1, and α‐SMA&SELENBP1 in the coronary tissue of both control and AS mice. Data are presented as mean ± standard deviation (SD). *p < 0.05, **p < 0.01. Scale bars are provided in the images.

Recognizing that the cellular composition of atherosclerotic plaques is crucial for mechanistic understanding, we further investigated hub genes expression within endothelial cells and VSMCs. IF colocalization demonstrated that CALCOCO2, TXNRD1, and SELENBP1 partially localized within CD31‐positive (endothelial) and α‐SMA positive (VSMC) regions, confirming their expression in these cell types (Figure 10A–F). Statistical analysis of IF costaining further showed a significant reduction in the number of double‐positive cells for all hub gene combinations with CD31 (CD31&CALCOCO2, CD31&TXNRD1, CD31&SELENBP1) and α‐SMA (α‐SMA&CALCOCO2, α‐SMA&TXNRD1, α‐SMA&SELENBP1) in AS group compared to the control group (Figure 10A–F). This finding indicates that the expression of all hub genes was significantly reduced in both endothelial cells and VSMCs within the coronary tissues of AS mice, consistent with the overall IHC findings. In summary, the expression of CALCOCO2, TXNRD1, and SELENBP1 was consistently and significantly downregulated in the coronary arteries of the AS mouse model.

## 4 DISCUSSION

This study identifies and validates CALCOCO2, TXNRD1, and SELENBP1 as novel biomarkers associated with ferroptosis and autophagy in AS. Through an integrated approach combining bioinformatics, machine learning, and in vivo models, we further characterize a distinct immune dysregulation signature in AS patients and reveal significant correlations between these hub genes and key immune cell populations, suggesting a link between these molecular signatures and the atherosclerotic immune microenvironment, providing a broader pathological context for these biomarkers.

Beyond the identification of individual hub genes, we first characterized the broader biological context from which these genes emerged. GO and KEGG enrichment analysis of the 104 common genes revealed significant enrichment in pathways related to lipid metabolism, redox regulation, and cytoskeletal dynamics—processes that directly underpin the core mechanisms of this study. For instance, enrichment in “fatty acid degradation” is particularly relevant to ferroptosis, as polyunsaturated fatty acids serve as the primary substrates for lipid peroxidation—the terminal execution step of this cell death pathway.^38^, ^39^ “Oxidation–reduction processes” are fundamental to maintaining cellular redox homeostasis, which plays a dual regulatory role in both ferroptosis and autophagy: on the one hand, GPX4 activity is central to suppressing ferroptosis by reducing lipid hydroperoxides^40^; on the other hand, ROS act as key signaling molecules in the initiation and regulation of autophagy.^41^ Redox imbalance is a known driver of endothelial dysfunction and inflammatory activation in AS.^41^, ^42^ Furthermore, “actin filament‐based processes” reflect cytoskeletal dynamics, which are critical for plaque stability: actin remodeling regulates VSMC migration (affecting fibrous cap thickness),^43^ endothelial barrier integrity (influencing monocyte infiltration),^44^ and foam cell motility. These processes are central to the maintenance of plaque integrity and the prevention of rupture.^45^ Collectively, these enriched pathways provide a broad biological foundation—encompassing lipid metabolism, redox regulation, and cytoskeletal integrity—that logically precedes and supports the subsequent focused analysis of CALCOCO2, TXNRD1, and SELENBP1 as hub genes linking ferroptosis and autophagy in AS.

CALCOCO2 (also known as NDP52) is a critical autophagy and mitophagy receptor that participates in selective clearance of damaged mitochondria and regulation of cellular redox balance.^46^, ^47^ Although recent studies have implicated CALCOCO2 in inflammatory responses and cardiovascular homeostasis,^48^, ^49^, ^50^ its specific role in the ferroptosis–autophagy crosstalk during atherosclerotic progression remains largely unexplored. In particular, no prior study has investigated CALCOCO2 as a hub gene connecting autophagy dysfunction and ferroptosis in AS. Our findings demonstrate that CALCOCO2 is significantly downregulated in AS plaques and exhibits strong diagnostic potential. As a crucial mitophagy receptor, CALCOCO2 is broadly implicated in diverse pathological conditions, including inflammatory disorders, multiple sclerosis, Crohn's disease, infectious diseases, sepsis, cancer, and cardiovascular diseases.^51^, ^52^, ^53^, ^54^, ^55^, ^56^, ^57^, ^58^ Regarding cardiovascular diseases, existing literature aligns with our findings: CALCOCO2 mitigates AngII‐induced atrial remodeling, and its deletion in macrophages impairs cholesterol efflux—a known pro‐atherogenic event.^58^, ^59^ Although these studies hinted at a protective role, the functional significance of CALCOCO2 within the specific context of AS plaques remained elusive. To further explore its functional implications, we performed GSEA based on CALCOCO2 expression levels. High CALCOCO2 expression was associated with pathways related to metabolic homeostasis and cellular quality control, whereas low expression was enriched in immune‐ and inflammation‐related pathways. These findings support a potential involvement of CALCOCO2 in ferroptosis and autophagy regulation, with the immune/inflammatory pathways likely representing secondary downstream phenotypes arising from impaired autophagy and dysregulated ferroptosis in atherosclerotic lesions, rather than primary functions of CALCOCO2 itself. Based on these observations and its established role in mitophagy,^46^, ^47^ we hypothesize that CALCOCO2 deficiency may impair mitophagy, potentially leading to unchecked mitochondrial ROS production and subsequent inflammation, which could collectively accelerate atherogenesis. Direct functional experiments are required to validate this proposed mechanism.

TXNRD1 is a key selenoprotein that maintains intracellular redox homeostasis and protects against oxidative stress–induced cell death.^60^ Emerging evidence indicates that TXNRD1 contributes to ferroptosis regulation by functioning in parallel with the GPX4/glutathione system to suppress lipid peroxidation.^61^, ^62^ Although TXNRD1 has been implicated in cardiovascular diseases,^63^, ^64^ its role in the crosstalk between autophagy and ferroptosis in atherosclerotic plaques has not been previously explored. Our study confronts the complex, and seemingly paradoxical, role of TXNRD1 in AS. Its diagnostic performance exhibited notable instability across independent datasets, which we attribute to the inherent heterogeneity of these cohorts. Specifically, GSE225650 compares atherosclerotic versus healthy coronary tissues,^24^ whereas GSE163154 compares IPH versus non‐IPH within advanced carotid plaques from symptomatic patients.^25^ These fundamental differences in tissue origin, disease stage, and classification criteria likely explain its divergent expression patterns. This substantial discrepancy—an AUC of 0.89 in GSE225650 versus 0.62 in GSE163154—fundamentally undermines the reliability of TXNRD1 as a stand‐alone diagnostic biomarker. However, rather than dismissing its relevance, this instability provides a critical clue: it points to a highly dynamic and context‐dependent function. TXNRD1 is broadly implicated in various biological processes, including cell phenotype regulation, proliferation, and signal transduction.^65^, ^66^ In terms of AS, the literature reflects this dichotomy: TXNRD1 is depicted as both a protective agent that mitigates macrophage oxidative stress and a detrimental factor that promotes pro‐inflammatory pathways in endothelial cells.^67^, ^68^ Our findings crystallize this paradox. We observed significantly lower expression of TXNRD1 in AS patient samples than in normal samples, yet found elevated levels in advanced, unstable intraplaque hemorrhage lesions. This downregulation in established disease was further mirrored in our AS mouse model. At first glance, the downregulation of TXNRD1 in the AS mouse model appears to conflict with its upregulation in human IPH lesions. However, this apparent discrepancy can be reconciled by considering the distinct pathological features of these models. The *Apoe* ^−/−^ mouse model on a high‐fat diet, while developing significant atherosclerotic lesions, does not recapitulate the IPH that characterizes the advanced, unstable plaques in the human IPH cohort.^25^ Therefore, the downregulation of TXNRD1 in the mouse model aligns with its decreased expression in the GSE225650 dataset (AS vs. normal) and likely reflects changes associated with plaque development in the absence of hemorrhage. In contrast, its upregulation specifically within the human IPH subgroup may represent a compensatory or maladaptive response to the extreme oxidative stress and tissue remodeling associated with plaque rupture and bleeding. To further explore its functional implications, we performed GSEA based on TXNRD1 expression levels. High TXNRD1 expression was notably enriched in the “regulation of autophagy” pathway, suggesting a potential link to autophagy regulation. Conversely, similar to CALCOCO2, low TXNRD1 expression was associated with immune/inflammatory pathway enrichment, which may represent inflammatory consequences of compromised antioxidant defense and imbalanced crosstalk between autophagy and ferroptosis. Based on these observations and its known dual roles reported in the literature,^67^, ^68^ we propose that the function of TXNRD1 in AS may be dynamic and context dependent, potentially switching from protective to detrimental as the disease progresses. This hypothesis, while consistent with our correlational data, requires direct experimental validation.

SELENBP1 is a selenium‐binding protein closely associated with redox regulation, oxidative stress response, and cellular detoxification.^69^ Recent studies suggest that SELENBP1 may affect ferroptosis by regulating GPX4 expression.^70^, ^71^ Although SELENBP1 has been linked to cardiovascular disease risk prediction^72^ and recently identified as a novel regulator of vascular remodeling in a VSMC subpopulation,^73^ its function in regulating autophagy–ferroptosis crosstalk in AS has not been reported. In our study, SELENBP1 showed strong diagnostic potential, with its expression consistently downregulated in both human atherosclerotic plaques and our AS mouse model—the first evidence of its dysregulation in this pathological context. Despite these compelling epidemiological and cellular links, a direct functional role for SELENBP1 within the atherosclerotic lesion itself has remained elusive. To further explore its functional implications, we performed GSEA based on SELENBP1 expression levels. Low SELENBP1 expression was associated with metabolic pathways closely linked to lipid peroxidation, redox balance, and autophagic activity. These pathways are all essential for ferroptosis execution and autophagy regulation, further supporting that SELENBP1 contributes to ferroptosis–autophagy crosstalk in AS. Based on these observations and its known role in selenium metabolism and redox regulation,^69^ we hypothesize that the loss of SELENBP1 may disrupt cellular redox homeostasis, potentially creating a pro‐inflammatory environment that accelerates plaque formation. Direct functional experiments are required to validate this proposed mechanism. Elucidating this specific mechanism is a critical next step and a promising direction for future research.

The colocalization of GPX4 and LC3 observed in our study provides direct morphological evidence for the functional crosstalk between ferroptosis and autophagy in AS, which is further supported by our key finding that both molecules are significantly downregulated in AS tissues (reflecting impaired autophagic flux and hyperactivated ferroptosis).^74^, ^75^ Although our observation of decreased LC3‐II expression suggests impaired autophagic flux, it is important to recognize that autophagy in AS is not exclusively protective. Depending on the cell type and pathological context, autophagy can also contribute to disease progression—as exemplified by the detrimental effects of dysregulated autophagy in VSMCs^15^ and macrophages.^16^, ^17^ In the context of our study, the concurrent downregulation of GPX4 and LC3 likely reflects a state of compromised cellular homeostasis in which both protective autophagy and ferroptosis defense mechanisms are overwhelmed. This interpretation aligns with the established role of basal autophagy as a survival mechanism in vascular cells,^76^ while acknowledging that more complex autophagic responses may occur in advanced plaques. Their concurrent downregulation and spatial coupling suggest that GPX4 and LC3 may coordinate the cross‐regulation of autophagy and ferroptosis in atherosclerotic lesions, and the disruption of this coordination may contributes to AS pathogenesis by forming a pathogenic vicious cycle. Based on our observations and existing literature, we speculate that this dysregulated crosstalk may involve a bidirectional regulatory loop: impaired autophagy may indirectly reduce the clearance of damaged organelles, which could potentially lead to increased ROS production^41^; this excessive ROS may further exacerbate lipid peroxide accumulation and repress GPX4 expression, thereby enhancing ferroptosis and amplifying vascular cell damage in atherosclerotic lesions.^77^ Conversely, hyperactivation of ferroptosis may feed back to inhibit autophagic activity, further exacerbating autophagic dysfunction.^18^ It should be noted that this potential regulatory loop is a reasonable hypothesis derived from our morphological and expression data, and the specific molecular details require further functional experiments to verify.

Beyond identifying hub genes, this study characterized the immune infiltration landscape of AS. We found that AS patients exhibited elevated naive B cells, plasma cells, M1 macrophages, and resting dendritic cells, alongside diminished resting CD4 memory T cells and NK cells. These findings are partly consistent with established pathology; the predominance of M1 macrophages in unstable plaques and the pro‐atherogenic role of antibody‐secreting plasma cells are well documented.^78^, ^79^ However, the immune context of AS is notoriously complex. The role of resting CD4 memory T cells, for instance, is highly debated. Our observation of their decrease aligns with some reports yet contradicts others showing an increase—a discrepancy likely attributable to differences in patient cohorts or disease staging.^80^, ^81^ Such contradictions suggest that the function of immune subsets in AS is not static but may be dynamic.

To directly link these immune alterations to our identified hub genes, we performed Spearman's correlation analysis between CALCOCO2, TXNRD1, and SELENBP1 expression and the relative abundance of 22 immune cell types in the GSE225650 dataset. Notably, all three hub genes showed significant negative correlations with M1 and M0 macrophages, and positive correlations with eosinophils, suggesting a shared role in suppressing pro‐inflammatory macrophage polarization and modulating eosinophil‐related immunity. Specifically, CALCOCO2 was strongly negatively correlated with M1 macrophages (*r* = −0.37, *p* < 0.0001) and positively correlated with resting memory CD4 T cells (*r* = 0.26, *p* < 0.01) and monocytes (*r* = 0.28, *p* < 0.001). These correlative findings suggest that CALCOCO2 may limit inflammatory signals by clearing damaged mitochondria and reducing oxidative stress, thereby influencing monocyte‐to‐macrophage differentiation and T‐cell activation.^46^, ^47^ TXNRD1, an antioxidant enzyme,^60^ exhibited broad immunomodulatory associations, including negative correlations with M1 macrophages (*r* = −0.36, *p* < 0.0001), naive B cells (*r* = −0.29, *p* < 0.001), and plasma cells (*r* = −0.25, *p* < 0.01), and positive correlations with resting memory CD4 T cells (*r* = 0.27, *p* < 0.01) and resting mast cells (*r* = 0.24, *p* < 0.01). These findings suggest that TXNRD1 may maintain immune homeostasis by suppressing both innate (macrophage) and adaptive (B‐cell) pro‐inflammatory responses, consistent with its role in redox balance.^60^ SELENBP1, a selenium‐binding protein,^69^ showed significant negative correlations with M1 macrophages (*r* = −0.27, *p* < 0.01) and activated memory CD4 T cells (*r* = −0.18, *p* < 0.05) and positive correlations with resting mast cells (*r* = 0.21, *p* < 0.05), suggesting a more focused role in restraining effector immune cell activation. Collectively, these correlations suggest that CALCOCO2, TXNRD1, and SELENBP1 may serve as immune‐associated hub genes that link molecular signatures to the immune microenvironment in AS, supporting their potential as therapeutic targets and immune modulators. These findings highlight the functional plasticity of the immune microenvironment in AS and suggest that dynamic immune monitoring may be necessary to develop effective, stage‐specific interventions.

We acknowledge several limitations that also frame the key directions for future research. First, the correlative nature of our key findings—including the associations between hub genes and immune cell populations—necessitates follow‐up studies to establish causal mechanisms. Although powerful for hypothesis generation, these correlations do not prove causation. Second, we did not validate the causal roles of CALCOCO2, TXNRD1, and SELENBP1 in regulating ferroptosis and autophagy pathways through direct functional experiments, such as gene knockdown or overexpression. Although our bioinformatic and correlative data suggest potential mechanistic roles—including the involvement of CALCOCO2 in mitophagy and SELENBP1 in redox homeostasis—these hypotheses require rigorous experimental validation in future studies. Third, although our findings were validated in a murine model, translating these results will require further investigation in human‐derived systems that more accurately recapitulate the complexity of human AS. Within this animal study, we also acknowledge that the relatively small sample size (*n* = 3 per group) may limit the statistical power and generalizability of these in vivo findings. Therefore, future animal studies with larger cohorts are warranted to validate these results and further explore the therapeutic potential of targeting these hub genes in AS. Fourth, although our GSEA and IF data support the involvement of CALCOCO2, TXNRD1, and SELENBP1 in ferroptosis and autophagy regulation, direct correlation analysis between hub gene expression and pathway markers (GPX4, LC3) was not performed in the current study. Future investigations should include such analyses to quantitatively validate these associations and further elucidate the regulatory relationships between these hub genes and the two pathways. Fifth, our promising biomarker data, derived from limited cohorts, underscores the need for validation in larger, multicenter prospective studies. Sixth, having identified these key genes, the critical next step is to dissect their underlying molecular pathways, a crucial step toward exploring their potential as therapeutic targets.

Our findings lay a clear foundation for future research. The foremost priority is to move beyond correlation and elucidate the causal molecular mechanisms of these hub genes—defining their upstream regulators, downstream effectors, and cell‐type‐specific functions in the vasculature. A parallel and critical direction is to investigate the crosstalk between these genes and key immune pathways, thereby unifying our molecular and immunological observations. To translate these discoveries, their diagnostic and prognostic value must be validated in large, prospective patient cohorts. Ultimately, this multipronged approach will be essential to formally establish these genes as viable therapeutic targets for AS.

In summary, our study identifies and validates CALCOCO2, TXNRD1, and SELENBP1 as hub genes associated with ferroptosis and autophagy in AS, and further reveals their significant correlations with key immune cell populations, linking these molecular signatures to the atherosclerotic immune microenvironment. These findings position them as promising candidate biomarkers and potential therapeutic targets for AS.

## 5 CONCLUSION

This study successfully identifies CALCOCO2, TXNRD1, and SELENBP1 as key hub genes implicated in AS through an integrated approach that combines bioinformatics analysis, machine learning methods, and animal experiments. Furthermore, our findings reveal significant alterations in immune cell profiles among patients with AS, indicating immune dysfunction and demonstrating that these hub genes are significantly correlated with multiple immune cell populations, thereby directly linking them to the atherosclerotic immune microenvironment. Collectively, these results provide novel insights into the pathogenesis of AS and offer candidate biomarkers and potential therapeutic targets for early diagnosis and intervention.

## AUTHOR CONTRIBUTIONS

**Xinou Zheng:** Conceptualization; data curation; formal analysis; methodology; software; writing – original draft. **Jinling Zheng:** Conceptualization; data curation; formal analysis; methodology; software. **Xuezhuang Li:** Validation. **Hua Chen:** Conceptualization; funding acquisition. **Li Zhang:** Conceptualization; data curation; formal analysis; methodology; writing – review and editing. **Yuqiong Zhao:** Conceptualization; data curation; formal analysis; methodology; writing – review and editing. **Yahao Ling:** Conceptualization; data curation; formal analysis; funding acquisition; software; writing – review and editing.

## FUNDING INFORMATION

This work was supported by the National Natural Science Foundation of China (no.: 32370568) in part by the Shenzhen Science and Technology Program (grant no.: JCYJ20220530165016037).

## CONFLICT OF INTEREST STATEMENT

The authors declare that they have no competing interests.

## ETHICS STATEMENT

All animal experiments were approved by and conducted in accordance with the guidelines of the Institutional Animal Care and Use Committee of Chinese PLA General Hospital (ID: 2023‐X19‐168).

## Supporting information


**Figure S1.** UMAP visualization of gene expression profiles from two GEO datasets (GSE22560 and GSE163154). (A) UMAP plot before batch effect removal, where samples cluster by dataset. (B) UMAP plot after batch effect correction using the removeBatchEffect function from the limma package, demonstrating improved integration of samples across datasets.
**Figure S2**. Functional enrichment analysis of the “orangered4” module genes. (A–C) GO enrichment analysis showing the top enriched terms in (A) Biological Process, (B) Cellular Component, and (C) Molecular Function. Dot size represents the number of genes enriched in each term, and color intensity indicates statistical significance expressed as‐log10(*p*‐value) (darker color represents higher significance). (D) KEGG pathway enrichment analysis of the “orangered4” module genes. Enrichment analyses were performed using the clusterProfiler package in R, with significance defined as‐log10(*p*‐value) ≥ 1.3 (corresponding to *p* ≤ 0.05).
**Figure S3**. 10‐fold cross‐validation repeated five times for hub genes. (A, B) Distribution of AUC values for CALCOCO2 in the GSE225650 and GSE163154 datasets, respectively, derived from five repetitions of 10‐fold cross‐validation. (C, D) Distribution of AUC values for TXNRD1 in the GSE225650 and GSE163154 datasets, respectively, derived from five repetitions of 10‐fold cross‐validation. (E, F) Distribution of AUC values for SELENBP1 in the GSE225650 and GSE163154 datasets, respectively, derived from five repetitions of 10‐fold cross‐validation. Red dots represent outliers.
**Figure S4**. Immune correlation analysis of hub genes in atherosclerosis. Spearman correlation analysis was performed between the expression levels of CALCOCO2, TXNRD1, and SELENBP1 and the relative abundance of 22 immune cell types estimated by CIBERSORT in the GSE225650 dataset. The heatmap displays correlation coefficients (*r*), with red indicating positive correlations and blue indicating negative correlations. Color intensity reflects the strength of the correlation. Statistical significance: *p* < 0.05, *p* < 0.01, *p* < 0.001, *p* < 0.0001 (corresponding to‐log10(*p*) ≥ 1.3, 2.0, 3.0, and 4.0, respectively).

## Data Availability

The datasets GSE225650 and GSE163154 analyzed during the current study are publicly available in the GEO database, accessible at https://www.ncbi.nlm.nih.gov/geo/. Further original data supporting the findings of this study are available from the corresponding author upon reasonable request.

## References

[1] Falk E . Pathogenesis of atherosclerosis. J Am Coll Cardiol. 2006;47(8 Suppl):C7‐C12. doi:10.1016/j.jacc.2005.09.068 16631513

[2] Williams JW , Winkels H , Durant CP , et al. Single cell RNA sequencing in atherosclerosis research. Circ Res. 2020;126(9):1112‐1126. doi:10.1161/circresaha.119.315940 32324494 PMC7185048

[3] Herrington W , Lacey B , Sherliker P , Armitage J , Lewington S . Epidemiology of atherosclerosis and the potential to reduce the global burden of atherothrombotic disease. Circ Res. 2016;118(4):535‐546. doi:10.1161/circresaha.115.307611 26892956

[4] World Heart Federation . World Heart Report 2023: Confronting the World's Number One Killer. Accessed December 10, 2023. https://heartreport23.world‐heart‐federation.org/

[5] Mushenkova NV , Summerhill VI , Zhang D , et al. Current advances in the diagnostic imaging of atherosclerosis: insights into the pathophysiology of vulnerable plaque. Int J Mol Sci. 2020;21(8):2992. doi:10.3390/ijms21082992 32340284 PMC7216001

[6] Revkin JH , Shear CL , Pouleur HG , Ryder SW , Orloff DG . Biomarkers in the prevention and treatment of atherosclerosis: need, validation, and future. Pharmacol Rev. 2007;59(1):40‐53. doi:10.1124/pr.59.1.1 17329547

[7] Dixon SJ , Lemberg KM , Lamprecht MR , et al. Ferroptosis: an iron‐dependent form of nonapoptotic cell death. Cell. 2012;149(5):1060‐1072. doi:10.1016/j.cell.2012.03.042 22632970 PMC3367386

[8] Guo Z , Ran Q , Roberts LJ , et al. Suppression of atherogenesis by overexpression of glutathione peroxidase‐4 in apolipoprotein E‐deficient mice. Free Radic Biol Med. 2008;44(3):343‐352. doi:10.1016/j.freeradbiomed.2007.09.009 18215741 PMC2245803

[9] Luo X , Wang Y , Zhu X , et al. MCL attenuates atherosclerosis by suppressing macrophage ferroptosis via targeting KEAP1/NRF2 interaction. Redox Biol. 2024;69:102987. doi:10.1016/j.redox.2023.102987 38100883 PMC10761782

[10] Hu M , Ladowski JM , Xu H . The role of autophagy in vascular endothelial cell health and physiology. Cells. 2024;13(10):825. doi:10.3390/cells13100825 38786047 PMC11120581

[11] Yu X , Zhang Y , Wang J , et al. Leonurine improves atherosclerosis by activating foam cell autophagy and metabolic remodeling via METTL3‐mediated AKT1S1 mRNA stability modulation. Phytomedicine. 2024;134:155939. doi:10.1016/j.phymed.2024.155939 39214016

[12] Bravo‐San Pedro JM , Kroemer G , Galluzzi L . Autophagy and mitophagy in cardiovascular disease. Circ Res. 2017;120(11):1812‐1824. doi:10.1161/circresaha.117.311082 28546358

[13] Grootaert MOJ , Moulis M , Roth L , et al. Vascular smooth muscle cell death, autophagy and senescence in atherosclerosis. Cardiovasc Res. 2018;114(4):622‐634. doi:10.1093/cvr/cvy007 29360955

[14] Shao BZ , Han BZ , Zeng YX , Su DF , Liu C . The roles of macrophage autophagy in atherosclerosis. Acta Pharmacol Sin. 2016;37(2):150‐156. doi:10.1038/aps.2015.87 26750103 PMC4753375

[15] Cheng CI , Lee YH , Chen PH , et al. Free fatty acids induce autophagy and LOX‐1 upregulation in cultured aortic vascular smooth muscle cells. J Cell Biochem. 2017;118(5):1249‐1261. doi:10.1002/jcb.25784 28072480

[16] Zhang Y , Vandestienne M , Lavillegrand JR , et al. Genetic inhibition of CARD9 accelerates the development of atherosclerosis in mice through CD36 dependent‐defective autophagy. Nat Commun. 2023;14(1):4622. doi:10.1038/s41467-023-40216-x 37528097 PMC10394049

[17] Li S , Zhou X , Duan Q , et al. Autophagy and its association with macrophages in clonal hematopoiesis leading to atherosclerosis. Int J Mol Sci. 2025;26(7):3252. doi:10.3390/ijms26073252 40244103 PMC11989900

[18] Yu W , Liu W , Xie D , et al. High level of uric acid promotes atherosclerosis by targeting NRF2‐mediated autophagy dysfunction and ferroptosis. Oxidative Med Cell Longev. 2022;2022:9304383. doi:10.1155/2022/9304383 PMC9038411 35480874

[19] Su G , Yang W , Wang S , Geng C , Guan X . SIRT1‐autophagy axis inhibits excess iron‐induced ferroptosis of foam cells and subsequently increases IL‐1Β and IL‐18. Biochem Biophys Res Commun. 2021;561:33‐39. doi:10.1016/j.bbrc.2021.05.011 34000515

[20] Hu G , Yuan Z , Wang J . Autophagy inhibition and ferroptosis activation during atherosclerosis: hypoxia‐inducible factor 1α inhibitor PX‐478 alleviates atherosclerosis by inducing autophagy and suppressing ferroptosis in macrophages. Biomed Pharmacother. 2023;161:114333. doi:10.1016/j.biopha.2023.114333 36948130

[21] Qiao L , Zhang X , Liu M , et al. Ginsenoside Rb1 enhances atherosclerotic plaque stability by improving autophagy and lipid metabolism in macrophage foam cells. Front Pharmacol. 2017;8:727. doi:10.3389/fphar.2017.00727 29114222 PMC5660703

[22] Niu H , Chen P , Fan L , Sun B . Comprehensive pan‐cancer analysis on CBX3 as a prognostic and immunological biomarker. BMC Med Genet. 2022;15(1):29. doi:10.1186/s12920-022-01179-y PMC8851738 35172803

[23] Nomiri S , Karami H , Baradaran B , et al. Exploiting systems biology to investigate the gene modules and drugs in ovarian cancer: a hypothesis based on the weighted gene co‐expression network analysis. Biomed Pharmacother. 2022;146:112537. doi:10.1016/j.biopha.2021.112537 34922114

[24] Mosquera JV , Auguste G , Wong D , et al. Integrative single‐cell meta‐analysis reveals disease‐relevant vascular cell states and markers in human atherosclerosis. Cell Rep. 2023;42(11):113380. doi:10.1016/j.celrep.2023.113380 37950869 PMC12335892

[25] Jin H , Goossens P , Juhasz P , et al. Integrative multiomics analysis of human atherosclerosis reveals a serum response factor‐driven network associated with intraplaque hemorrhage. Clin Transl Med. 2021;11(6):e458. doi:10.1002/ctm2.458 34185408 PMC8236116

[26] Taminau J , Meganck S , Lazar C , et al. Unlocking the potential of publicly available microarray data using inSilicoDb and inSilicoMerging R/Bioconductor packages. BMC Bioinformatics. 2012;13:335. doi:10.1186/1471-2105-13-335 23259851 PMC3568420

[27] Ritchie ME , Phipson B , Wu D , et al. Limma powers differential expression analyses for RNA‐sequencing and microarray studies. Nucleic Acids Res. 2015;43(7):e47. doi:10.1093/nar/gkv007 25605792 PMC4402510

[28] He X , Zhang J , Gong M , et al. Identification of potential ferroptosis‐associated biomarkers in rheumatoid arthritis. Front Immunol. 2023;14:1197275. doi:10.3389/fimmu.2023.1197275 37492576 PMC10364059

[29] Xu W , Su X , Qin J , et al. Identification of autophagy‐related biomarkers and diagnostic model in Alzheimer's disease. Gene (Basel). 2024;15(8):1027. doi:10.3390/genes15081027 PMC11354206 39202387

[30] Cui K , Li Z . Identification and analysis of type 2 diabetes‐mellitus‐associated autophagy‐related genes. Front Endocrinol (Lausanne). 2023;14:1164112. doi:10.3389/fendo.2023.1164112 37223013 PMC10200926

[31] Langfelder P , Horvath S . WGCNA: an R package for weighted correlation network analysis. BMC Bioinformatics. 2008;9:559. doi:10.1186/1471-2105-9-559 19114008 PMC2631488

[32] Yu G , Wang LG , Han Y , He QY . clusterProfiler: an R package for comparing biological themes among gene clusters. OMICS. 2012;16(5):284‐287. doi:10.1089/omi.2011.0118 22455463 PMC3339379

[33] Newman AM , Liu CL , Green MR , et al. Robust enumeration of cell subsets from tissue expression profiles. Nat Methods. 2015;12(5):453‐457. doi:10.1038/nmeth.3337 25822800 PMC4739640

[34] Brehm ZP , Sherina V , Rosenberg AZ , Halushka MK , Mccall MN . Considerations for deconvolution: a case study with GTEx coronary artery tissues. bioRxiv. 2022:2022.05.17.492324. doi:10.1101/2022.05.17.492324

[35] Gao J , Shi L , Gu J , et al. Difference of immune cell infiltration between stable and unstable carotid artery atherosclerosis. J Cell Mol Med. 2021;25(23):10973‐10979. doi: 10.1111/jcmm.17018 34729909 PMC8642673

[36] Xu Y , Su G‐H , Ma D , et al. Technological advances in cancer immunity: from immunogenomics to single‐cell analysis and artificial intelligence. Signal Transduct Target Ther. 2021;6(1):312. doi:10.1038/s41392-021-00729-7 34417437 PMC8377461

[37] Zhu Y , Yang J , Zhang JL , et al. Dapagliflozin activates the RAP1B/NRF2/GPX4 signaling and promotes mitochondrial biogenesis to alleviate vascular endothelial ferroptosis. Cell Signal. 2025;132:111824. doi:10.1016/j.cellsig.2025.111824 40280228

[38] Noguchi N , Saito Y , Niki E . Lipid peroxidation, ferroptosis, and antioxidants. Free Radic Biol Med. 2025;237:228‐238. doi:10.1016/j.freeradbiomed.2025.05.393 40374017

[39] Lee J , Roh JL . Lipid metabolism in ferroptosis: unraveling key mechanisms and therapeutic potential in cancer. Biochim Biophys Acta Rev Cancer. 2025;1880(1):189258. doi:10.1016/j.bbcan.2024.189258 39746458

[40] Su LJ , Zhang JH , Gomez H , et al. Reactive oxygen species‐induced lipid peroxidation in apoptosis, autophagy, and ferroptosis. Oxidative Med Cell Longev. 2019;2019:5080843. doi:10.1155/2019/5080843 PMC6815535 31737171

[41] Jeong SJ , Oh GT . Unbalanced redox with autophagy in cardiovascular disease. J Lipid Atheroscler. 2023;12(2):132‐151. doi:10.12997/jla.2023.12.2.132 37265853 PMC10232220

[42] Penna C , Pagliaro P . Endothelial dysfunction: redox imbalance, NLRP3 inflammasome, and inflammatory responses in cardiovascular diseases. Antioxidants (Basel). 2025;14(3):256. doi:10.3390/antiox14030256 40227195 PMC11939635

[43] Lyle AN , Taylor WR . RACing up a new regulatory mechanism for vascular smooth muscle cell migration. Arterioscler Thromb Vasc Biol. 2013;33(4):667‐669. doi:10.1161/ATVBAHA.13.301022 23486767

[44] Hashimoto K , Kataoka N , Nakamura E , Tsujioka K , Kajiya F . Oxidized LDL specifically promotes the initiation of monocyte invasion during transendothelial migration with upregulated PECAM‐1 and downregulated VE‐cadherin on endothelial junctions. Atherosclerosis. 2007;194(2):e9‐e17. doi:10.1016/j.atherosclerosis.2006.11.029 17194459

[45] Garcia‐Arguinzonis M , Escate R , Lugano R , et al. Gene expression pattern associated with cytoskeletal remodeling in lipid‐loaded human vascular smooth muscle cells: crosstalk between C3 complement and the focal adhesion protein paxillin. Cells. 2025;14(16):1245. doi:10.3390/cells14161245 40862724 PMC12384099

[46] Bruqi K , Strappazzon F . NDP52 and its emerging role in pathogenesis. Cell Death Dis. 2025;16(1):359. doi:10.1038/s41419-025-07668-z 40319017 PMC12049512

[47] Jo C , Gundemir S , Pritchard S , et al. Nrf2 reduces levels of phosphorylated tau protein by inducing autophagy adaptor protein NDP52. Nat Commun. 2014;5:3496. doi:10.1038/ncomms4496 24667209 PMC3990284

[48] Robinson CA , Singh GK , Kleer M , et al. Kaposi's sarcoma‐associated herpesvirus (KSHV) utilizes the NDP52/CALCOCO2 selective autophagy receptor to disassemble processing bodies. PLoS Pathog. 2023;19(1):e1011080. doi:10.1371/journal.ppat.1011080 36634147 PMC9876383

[49] Gil‐Cayuela C , López A , Martínez‐Dolz L , et al. The altered expression of autophagy‐related genes participates in heart failure: NRBP2 and CALCOCO2 are associated with left ventricular dysfunction parameters in human dilated cardiomyopathy. PLoS One. 2019;14(4):e0215818. doi:10.1371/journal.pone.0215818 31009519 PMC6476534

[50] Gao A , Wang M , Tang X , et al. NDP52 SUMOylation contributes to low‐dose X‐rays‐induced cardiac hypertrophy through PINK1/Parkin‐mediated mitophagy via MUL1/SUMO2 signalling. J Cell Physiol. 2024;239(1):79‐96. doi:10.1002/jcp.31145 37942585

[51] Heo JM , Ordureau A , Paulo JA , Rinehart J , Harper JW . The PINK1‐PARKIN mitochondrial ubiquitylation pathway drives a program of OPTN/NDP52 recruitment and TBK1 activation to promote mitophagy. Mol Cell. 2015;60(1):7‐20. doi:10.1016/j.molcel.2015.08.016 26365381 PMC4592482

[52] Di Rita A , Strappazzon F . A protective variant of the autophagy receptor CALCOCO2/NDP52 in multiple sclerosis (MS). Autophagy. 2021;17(6):1565‐1567. doi:10.1080/15548627.2021.1924969 33970776 PMC8205020

[53] Till A , Lipinski S , Ellinghaus D , et al. Autophagy receptor CALCOCO2/NDP52 takes center stage in Crohn disease. Autophagy. 2013;9(8):1256‐1257. doi:10.4161/auto.25483 23820297 PMC3748200

[54] Leymarie O , Meyer L , Tafforeau L , et al. Influenza virus protein PB1‐F2 interacts with CALCOCO2 (NDP52) to modulate innate immune response. J Gen Virol. 2017;98(6):1196‐1208. doi:10.1099/jgv.0.000782 28613140

[55] Petkova DS , Verlhac P , Rozières A , et al. Distinct contributions of autophagy receptors in measles virus replication. Viruses. 2017;9(5):123. doi:10.3390/v9050123 28531150 PMC5454435

[56] Lu J , Zhang J , Jiang H , et al. Vangl2 suppresses NF‐κB signaling and ameliorates sepsis by targeting p65 for NDP52‐mediated autophagic degradation. elife. 2024;12:RP87935. doi:10.7554/eLife.87935 39269442 PMC11398866

[57] Cui F , Wang S , Tan J , et al. Calcium‐binding and coiled‐coil domain 2 promotes the proliferation and suppresses apoptosis of prostate cancer cells. Exp Ther Med. 2021;21(4):405. doi:10.3892/etm.2021.9836 33692836 PMC7938445

[58] Sang W , Yan X , Wang L , et al. CALCOCO2 prevents AngII‐induced atrial remodeling by regulating the interaction between mitophagy and mitochondrial stress. Int Immunopharmacol. 2024;140:112841. doi:10.1016/j.intimp.2024.112841 39094358

[59] Robichaud S , Fairman G , Vijithakumar V , et al. Identification of novel lipid droplet factors that regulate lipophagy and cholesterol efflux in macrophage foam cells. Autophagy. 2021;17(11):3671‐3689. doi:10.1080/15548627.2021.1886839 33590792 PMC8632324

[60] Arnér ESJ . Targeting the selenoprotein thioredoxin reductase 1 for anticancer therapy. Adv Cancer Res. 2017;136:139‐151. doi:10.1016/bs.acr.2017.07.005 29054416

[61] Cheff DM , Huang C , Scholzen KC , et al. The ferroptosis inducing compounds RSL3 and ML162 are not direct inhibitors of GPX4 but of TXNRD1. Redox Biol. 2023;62:102703. doi:10.1016/j.redox.2023.102703 37087975 PMC10149367

[62] Su C , Xue Y , Fan S , et al. Ferroptosis and its relationship with cancer. Front Cell Dev Biol. 2024;12:1423869. doi:10.3389/fcell.2024.1423869 39877159 PMC11772186

[63] Rose AH , Hoffmann PR . Selenoproteins and cardiovascular stress. Thromb Haemost. 2015;113(3):494‐504. doi:10.1160/th14-07-0603 25354851 PMC5705008

[64] Rocca C , Pasqua T , Boukhzar L , Anouar Y , Angelone T . Progress in the emerging role of selenoproteins in cardiovascular disease: focus on endoplasmic reticulum‐resident selenoproteins. Cell Mol Life Sci. 2019;76(20):3969‐3985. doi:10.1007/s00018-019-03195-1 31218451 PMC11105271

[65] Carlson BA , Yoo MH , Tobe R , et al. Thioredoxin reductase 1 protects against chemically induced hepatocarcinogenesis via control of cellular redox homeostasis. Carcinogenesis. 2012;33(9):1806‐1813. doi:10.1093/carcin/bgs230 22791808 PMC3514905

[66] Rundlöf AK , Arnér ES . Regulation of the mammalian selenoprotein thioredoxin reductase 1 in relation to cellular phenotype, growth, and signaling events. Antioxid Redox Signal. 2004;6(1):41‐52. doi:10.1089/152308604771978336 14980055

[67] Hägg D , Englund MC , Jernås M , et al. Oxidized LDL induces a coordinated up‐regulation of the glutathione and thioredoxin systems in human macrophages. Atherosclerosis. 2006;185(2):282‐289. doi:10.1016/j.atherosclerosis.2005.06.034 16046214

[68] Liu ZB , Shen X . Thioredoxin reductase 1 upregulates MCP‐1 release in human endothelial cells. Biochem Biophys Res Commun. 2009;386(4):703‐708. doi:10.1016/j.bbrc.2009.06.100 19555664

[69] Köhnlein K , Urban N , Guerrero‐Gómez D , et al. A *Caenorhabditis elegans* ortholog of human selenium‐binding protein 1 is a pro‐aging factor protecting against selenite toxicity. Redox Biol. 2020;28:101323. doi:10.1016/j.redox.2019.101323 31557719 PMC6812014

[70] Zhao W , Nikolic‐Paterson DJ , Li K , et al. Selenium binding protein 1 protects renal tubular epithelial cells from ferroptosis by upregulating glutathione peroxidase 4. Chem Biol Interact. 2024;393:110944. doi:10.1016/j.cbi.2024.110944 38518851

[71] Ma J , Li Z , Xu J , et al. PRDM1 promotes the ferroptosis and immune escape of thyroid cancer by regulating USP15‐mediated SELENBP1 deubiquitination. J Endocrinol Investig. 2024;47(12):2981‐2997. doi:10.1007/s40618-024-02385-4 39014173

[72] Kühn EC , Slagman A , Kühn‐Heid ECD , et al. Circulating levels of selenium‐binding protein 1 (SELENBP1) are associated with risk for major adverse cardiac events and death. J Trace Elem Med Biol. 2019;52:247‐253. doi:10.1016/j.jtemb.2019.01.005 30732890

[73] Cai C , Weng Y , Wang X , et al. Single‐cell RNA landscape of cell heterogeneity and immune microenvironment in ligation‐induced vascular remodeling in rat. Atherosclerosis. 2023;377:1‐11. doi:10.1016/j.atherosclerosis.2023.06.010 37343431

[74] Zhang J , Wang X , Guan B , et al. Qing‐Xin‐Jie‐Yu granule inhibits ferroptosis and stabilizes atherosclerotic plaques by regulating the GPX4/xCT signaling pathway. J Ethnopharmacol. 2023;301:115852. doi:10.1016/j.jep.2022.115852 36272494

[75] Peng Q , Liu H , Luo Z , et al. Effect of autophagy on ferroptosis in foam cells via Nrf2. Mol Cell Biochem. 2022;477(5):1597‐1606. doi:10.1007/s11010-021-04347-3 35195807

[76] De Meyer GR , Grootaert MO , Michiels CF , et al. Autophagy in vascular disease. Circ Res. 2015;116(3):468‐479. doi:10.1161/circresaha.116.303804 25634970

[77] Wu S , Liang Y , Weng Y , et al. Study on the effect and mechanism of fraxetin on atherosclerosis based on ferroptosis regulation. J Guangxi Med Univ. 2023;40(5):716‐723. doi:10.16190/j.cnki.45-1211/r.2023.05.002

[78] Momtazi‐Borojeni AA , Abdollahi E , Nikfar B , Chaichian S , Ekhlasi‐Hundrieser M . Curcumin as a potential modulator of M1 and M2 macrophages: new insights in atherosclerosis therapy. Heart Fail Rev. 2019;24(3):399‐409. doi:10.1007/s10741-018-09764-z 30673930

[79] Tay C , Liu YH , Kanellakis P , et al. Follicular B cells promote atherosclerosis via T cell‐mediated differentiation into plasma cells and secreting pathogenic immunoglobulin G. Arterioscler Thromb Vasc Biol. 2018;38(5):e71‐e84. doi:10.1161/atvbaha.117.310678 29599140

[80] Zheng Z , Yuan D , Shen C , et al. Identification of potential diagnostic biomarkers of atherosclerosis based on bioinformatics strategy. BMC Med Genet. 2023;16(1):100. doi:10.1186/s12920-023-01531-w PMC10176947 37173673

[81] Zheng Z , Li K , Yang Z , et al. Transcriptomic analysis reveals molecular characterization and immune landscape of PANoptosis‐related genes in atherosclerosis. Inflamm Res. 2024;73(6):961‐978. doi:10.1007/s00011-024-01877-6 38587531

