# Supplementary material for: Multi‐omics data mining combined with experimental validation reveals ferroptosis‐ and autophagy‐associated hub genes as diagnostic candidates and immune modulators in atherosclerosis
Source: Animal Model Exp Med. 2026 May 6;9(4):652–73. doi: 10.1002/ame2.70208 (PMC13242735; doi:10.1002/ame2.70208)
Supplement: Supplementary file 1 — Figure S1. UMAP visualization of gene expression profiles from two GEO datasets (GSE22560 and GSE163154). (A) UMAP plot before batch effect removal, where samples cluster by dataset. (B) UMAP plot after batch effect correction using the removeBatchEffect function from the limma package, demonstrating improved integration of samples across datasets. Figure S2. Functional enrichment analysis of the “orangered4” module genes. (A–C) GO enrichment analysis showing the top enriched terms in (A) Biological Process, (B) Cellular Component, and (C) Molecular Function. Dot size represents the number of genes enriched in each term, and color intensity indicates statistical significance expressed as‐log10(p‐value) (darker color represents higher significance). (D) KEGG pathway enrichment analysis of the “orangered4” module genes. Enrichment analyses were performed using the clusterProfiler package in R, with significance defined as‐log10(p‐value) ≥ 1.3 (corresponding to p ≤ 0.05). Figure S3. 10‐fold cross‐validation repeated five times for hub genes. (A, B) Distribution of AUC values for CALCOCO2 in the GSE225650 and GSE163154 datasets, respectively, derived from five repetitions of 10‐fold cross‐validation. (C, D) Distribution of AUC values for TXNRD1 in the GSE225650 and GSE163154 datasets, respectively, derived from five repetitions of 10‐fold cross‐validation. (E, F) Distribution of AUC values for SELENBP1 in the GSE225650 and GSE163154 datasets, respectively, derived from five repetitions of 10‐fold cross‐validation. Red dots represent outliers. Figure S4. Immune correlation analysis of hub genes in atherosclerosis. Spearman correlation analysis was performed between the expression levels of CALCOCO2, TXNRD1, and SELENBP1 and the relative abundance of 22 immune cell types estimated by CIBERSORT in the GSE225650 dataset. The heatmap displays correlation coefficients (r), with red indicating positive correlations and blue indicating negative correlations. Color intensity ref [file AME2-9-652-s001.pdf]

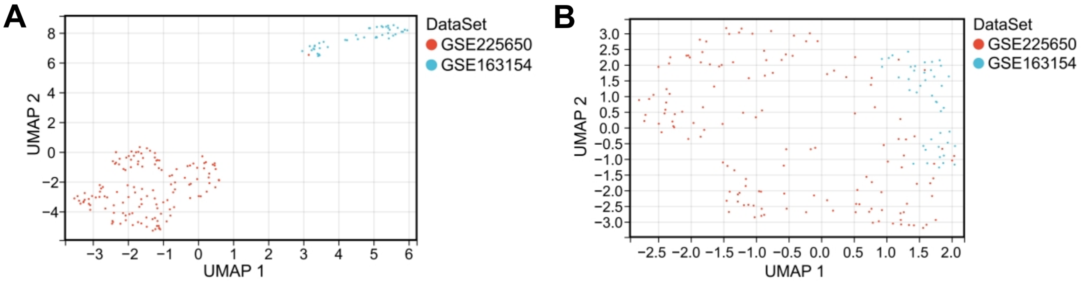

Figure S1. UMAP visualization of gene expression profiles from two GEO datasets (GSE22560 and GSE163154). (A) UMAP plot before batch effect removal, where samples cluster by dataset. (B) UMAP plot after batch effect correction using the removeBatchEffect function from the limma package, demonstrating improved integration of samples across datasets.

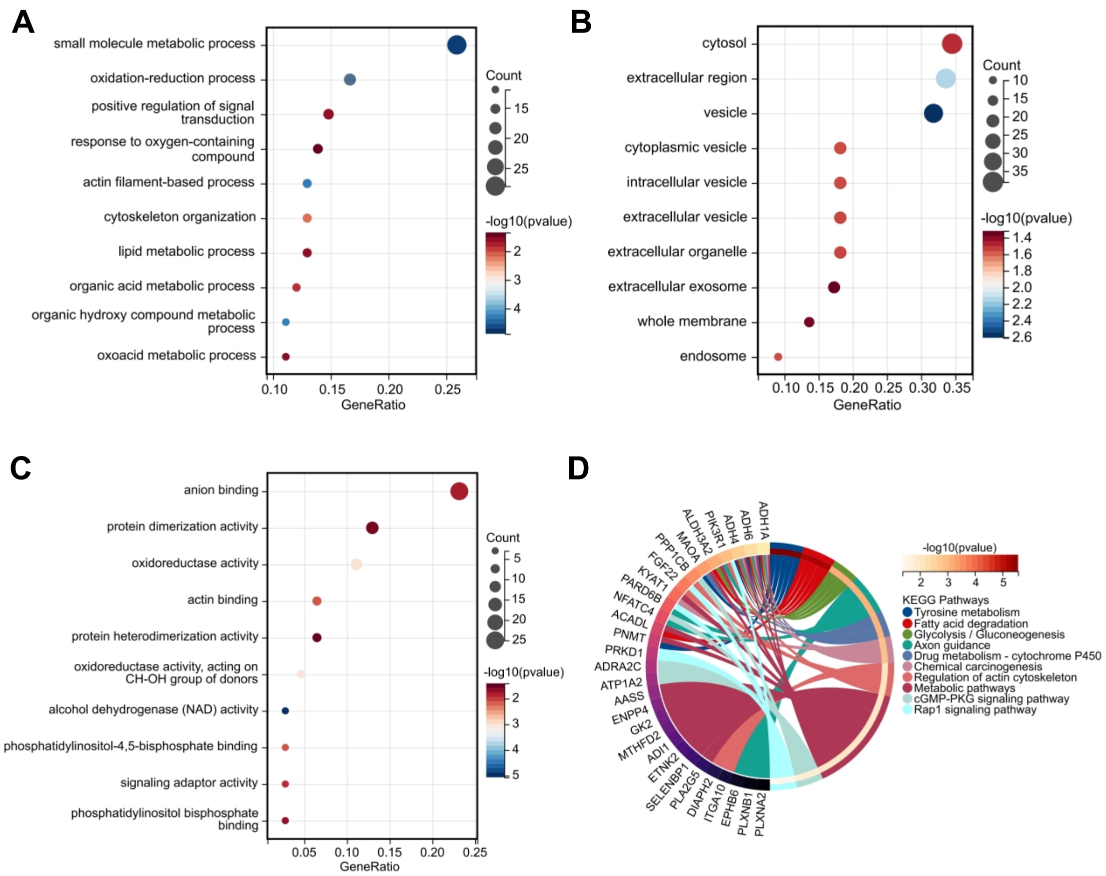

Figure S2. Functional enrichment analysis of the "orangered4" module genes. (A-C) GO enrichment analysis showing the top enriched terms in (A) Biological Process, (B) Cellular Component, and (C) Molecular Function. Dot size represents the number of genes enriched in each term, and color intensity indicates statistical significance expressed as -log10(p-value) (darker color represents higher significance). (D) KEGG pathway enrichment analysis of the "orangered4" module genes. Enrichment analyses were performed using the clusterProfiler package in R, with significance defined as -log10(p-value) $\geq$ 1.3 (corresponding to $p \leq 0.05$).

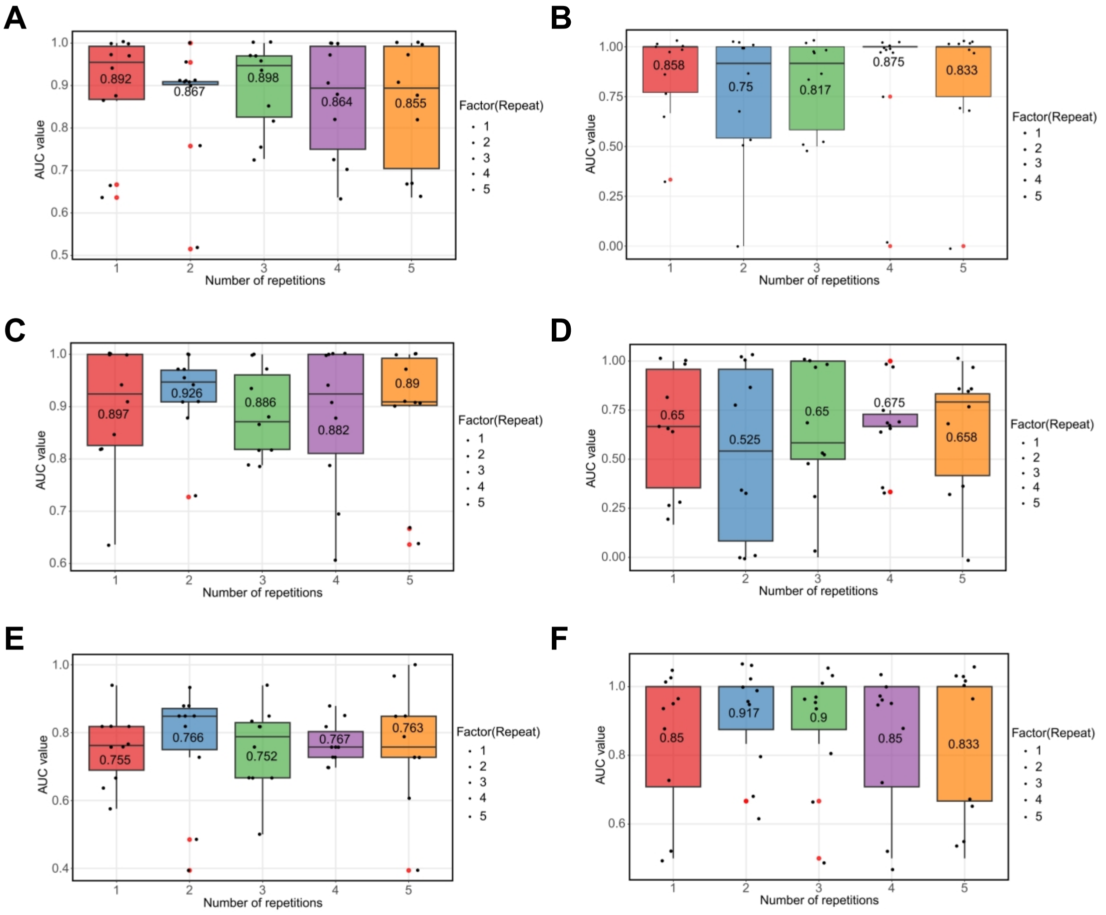

Figure S3. 10-fold cross-validation repeated five times for hub genes. (A, B) Distribution of AUC values for CALCOCO2 in the GSE225650 and GSE163154 datasets, respectively, derived from five repetitions of 10-fold cross-validation. (C, D) Distribution of AUC values for TXNRD1 in the GSE225650 and GSE163154 datasets, respectively, derived from five repetitions of 10-fold cross-validation. (E, F) Distribution of AUC values for SELENBP1 in the GSE225650 and GSE163154 datasets, respectively, derived from five repetitions of 10-fold cross-validation. Red dots represent outliers.

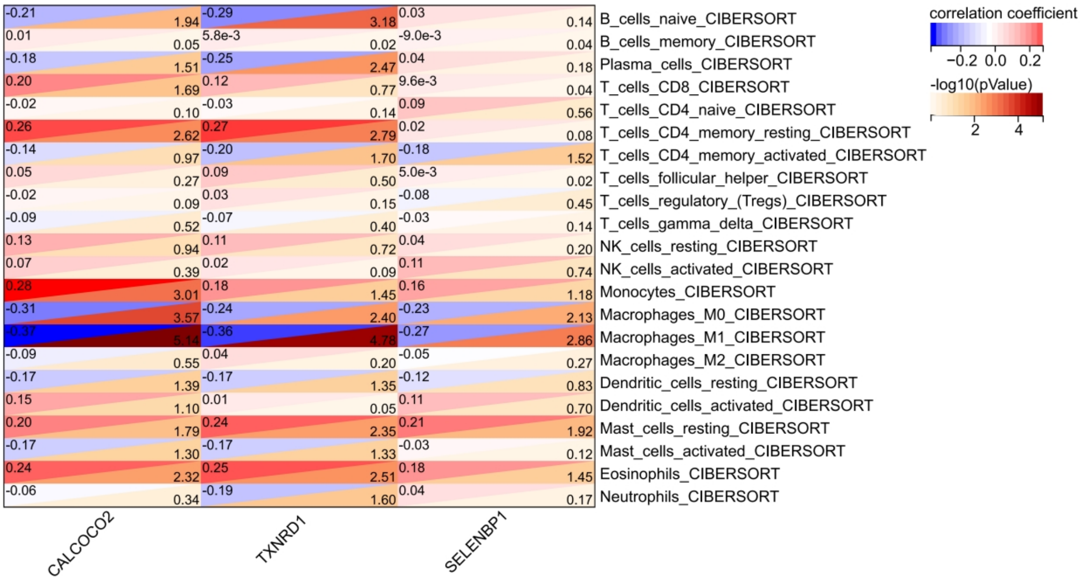

Figure S4. Immune correlation analysis of hub genes in atherosclerosis. Spearman correlation analysis was performed between the expression levels of CALCOCO2, TXNRD1, and SELENBP1 and the relative abundance of 22 immune cell types estimated by CIBERSORT in the GSE225650 dataset. The heatmap displays correlation coefficients (r), with red indicating positive correlations and blue indicating negative correlations. Color intensity reflects the strength of the correlation. Statistical significance: $p < 0.05$, $p < 0.01$, $p < 0.001$, $p < 0.0001$ (corresponding to -log10(p) ⩾ 1.3, 2.0, 3.0, and 4.0, respectively).
